# NUMB maintains bone mass by promoting degradation of PTEN and GLI1 via ubiquitination in osteoblasts

**DOI:** 10.1038/s41413-018-0030-y

**Published:** 2018-11-10

**Authors:** Ling Ye, Feng Lou, Fanyuan Yu, Demao Zhang, Chenglin Wang, Fanzi Wu, Xin Li, Yilin Ping, Xiao Yang, Jing Yang, Dian Chen, Bo Gao, Dingming Huang, Peng Liu

**Affiliations:** 1 State Key Laboratory of Oral Disease, West China Hospital of Stomatology, Sichuan University, Chengdu, China; 2 The Mount Sinai Bone Program, Department of Medicine, Mount Sinai School of Medicine, New York City, NY 10029 USA

## Abstract

The adaptor protein NUMB is involved in asymmetric division and cell fate determination and recognized as an antagonist of Notch. Previous studies have proved that Notch activation in osteoblasts contributes to a high bone mass. In this study,  however, an osteopenic phenotype was found in 9-week-old mice using osteoblastic specific *Col1a1–2.3-Cre* to ablate both *Numb* and its homologue *Numbl* . The trabecular bone mass decreased dramatically while the cortical bone mass was unaffected. Here, the Notch signal was not activated, while the tensin homologue deleted on human chromosome 10 (PTEN), which dephosphorylates phosphatidylinositide 3-kinases, was elevated, attenuating protein kinase B (Akt). The ubiquitination assay revealed that NUMB may physiologically promote PTEN ubiquitination in the presence of neural precursor cell-expressed developmentally downregulated protein 4–1. In addition, the deficiency of *Numb*/*Numbl* also activated the Hedgehog pathway through GLI1. This process was found to improve the ratio of the receptor activator of nuclear factor-kB ligand to osteoprotegerin, which enhanced the differentiation of osteoclasts and bone resorption . In conclusion, this study provides an insight into  new functons of   NUMB and NUMBL on bone homeostasis.

## Homeostasis: Protein regulators of bone mass

The related proteins NUMB and NUMBL maintain the survival of bone-generating osteoblast cells. NUMB was previously recognized to antagonize Notch signaling pathway ; In this study, it observes  that genetically altered mice, unable to express the two proteins, suffered from degraded bone quality. This suggests that the two proteins play a more complex, nuanced role in the process of bone mass maintenance. The team’s studies showed that NUMB and NUMBL suppression inhibits a signaling pathway important to skeletal development and protein synthesis in osteoblasts, though raise that further investigations are essential to elucidate the exact mechanistic action of these proteins. The authors of this study suggest that NUMB constitutes a potential target for therapies targeting bone loss and reduced bone strength in patients with osteoporosis.

## Introduction

Osteoporosis (OP) is an age-related disease. Dysfunction in bone remodelling leads to osteopenia and enhances skeletal fragility^1^. In adult bones, osteoblasts participate in bone remodelling by coupling with bone-resorbing osteoclasts^2,3^. They regulate bone resorption by stimulating osteoclast differentiation via producing monocyte colony-stimulating factor, receptor activator of nuclear factor kB ligand (RANKL) and osteoprotegerin (OPG)^4,5^. Coordination between osteoblasts and osteoclasts is vital for maintaining the adult bone mass. Disruption of this balance has been found in many diseases, such as OP, rheumatoid arthritis, and Paget’s disease^6,7^. In osteoblasts, exquisite and delicate molecular signals are involved in regulating this balance^8^. Notch and Hedgehog (Hh) are essential signals. In vivo, a gain of a Notch signal in osteoblasts accelerates the proliferation of immature osteoblasts causing osteosclerosis, while the loss of a Notch signal in osteoblasts gives rise to age-related OP associated with enhanced osteoclastic activity^9^. Abnormal activation of Hh in mature osteoblasts causes excessive bone resorption. Increased Hh promotes the secretion of RANKL and the differentiation of osteoclasts, thus  causes severe bone loss^10^. Hence, alterations in osteoblasts not only influence the behaviour of the osteoblasts themselves but also regulate osteoclastogenesis via a RANKL-dependent pattern to affect the osteoblast–osteoclast balance.

NUMB, a membrane-associated protein encoded by the *Numb* gene, was initially identified as d-numb in sensory organ precursors of *Drosophila melanogaster*. In the central nervous system of *Drosophila*, d-numb is implicated in cell fate determination as an antagonist of Notch^11–13^. In *Mus musculus*, *Numb* has a homologue *Numbl* and encodes four spliced transcripts (NUMB1–4)^14^. NUMB is believed to regulate neurogenesis, myogenesis, and tumourigenesis in Notch-dependent or Notch-independent ways^15–21^. Previous studies suggested that NUMB antagonized canonical Notch in osteoclasts^22^. An increase in NUMBL and a decrease in the level of the Notch intracellular domain (NICD) mediate the production of osteopetrosis via impaired osteoclastogenesis in tumour growth factor-β–activated kinase 1–deficient mice^23^. These studies suggested that NUMB/NUMBL might be a potential regulatory factor for bone mass. However, no studies have yet  determined whether the expression of NUMB/NUMBL in osteoblast lineage cells regulates the coordination between osteoblasts and osteoclasts.

This study demonstrated that NUMB in osteoblasts contributed to bone mass independently of Notch  pathway. The double-knockout of *Numb* and *Numbl* leads to the accumulation of phosphatase and tensin homologue deleted on human chromosome 10 (PTEN) and suppresses osteoblast survival. The underlying mechanism was an increase in PTEN, resulting in osteoblast-impaired protein kinase B–mechanistic target of rapamycin (Akt-mTOR) signalling, which has been widely reported to modulate the proliferation, apoptosis, and differentiation of osteoblasts^24–27^. Moreover, the  decreased expression of *Numb* and *Numbl* in osteoblasts  caused to the upregulation of the GLI1 level, which in turn boosted the ratio of RANKL/OPG and subsequently enhanced bone resorption. Thus, this study not only clarified the pivotal role of NUMB and NUMBL in bone homeostasis but also suggested novel functions of specific ubiquitination in the fine-tuning of bone homeostasis.

## Results

### Knockout of *Numb*/*Numbl* in osteoblasts caused OP

Primary BMSCs from C57BL/6J mice were induced to osteoblasts to understand the temporal-spatial  expression of NUMB protein in osteogenesis. The wells were stained using an anti-NUMB antibody (with possible cross-reaction with NUMBL) on day 15, when the calcification of the cell colonies had begun. The red NUMB-positive signal mainly clustered at the periphery (black arrow) or the centre (yellow arrow) of the mineralizing nodule (red arrow) (Supplementary Fig. S1a). A noticeable  increase in the expression of *Numb* and *Numbl* occurred during osteogenesis (Supplementary Fig. S1b). At day 21, the expression of *Numb* was 5.13 times higher than the expression of *Numbl* (Supplementary Fig. S1c). Western Blot data also showed continually increasing NUMB protein expression along with osteoblastic differentiation (Supplementary Fig. S1d, e). The results indicated that NUMB was highly expressed in osteoblasts.

A progressive increase in NUMB expression in osteoblastic lineage cells suggests the potential importance of NUMB in osteoblasts. The expression of *Numb* and *Numbl* was eliminated by crossing N/Nl-floxed mice with a well-defined line of Col2.3-Cre that expressed Cre recombinase in mature osteoblasts and premature osteocytes^28^. Here, 2.3-Cre; N/Nl-floxed mice were used as DKOs, while N/NL-floxed littermates were used as WT controls. Frozen sections of distal femurs from WT and DKO mice were stained with anti-NUMB to examine the expression of *Numb* in vivo (Supplementary Fig. S2). In WTs, NUMB proteins were expressed  mainly  on the trabecular and cortical bone surfaces (black arrows), while  a few  were expressed in the bone marrow nearby (red arrows). As reported earlier, these signals originated in the dominant haemopoietic cells in the bone marrow^29^. In DKO mice, the majority of NUMB on the bone surface was deleted by Col2.3-Cre, leaving small amounts possibly linked to osteoclastic lineage cells, as described in previous studies^22,23^. The positive area in the bone marrow of the DKO mice did not change significantly (red arrows in DKOs). The efficiency of excision of *Numb* and *Numbl* was confirmed by qRT-PCR as 76.65% and 44.04%, respectively (Supplementary Fig. S3a). Thus, the gene knockouts were effective and  specifically performed  in osteoblasts.

**Fig. 1:**
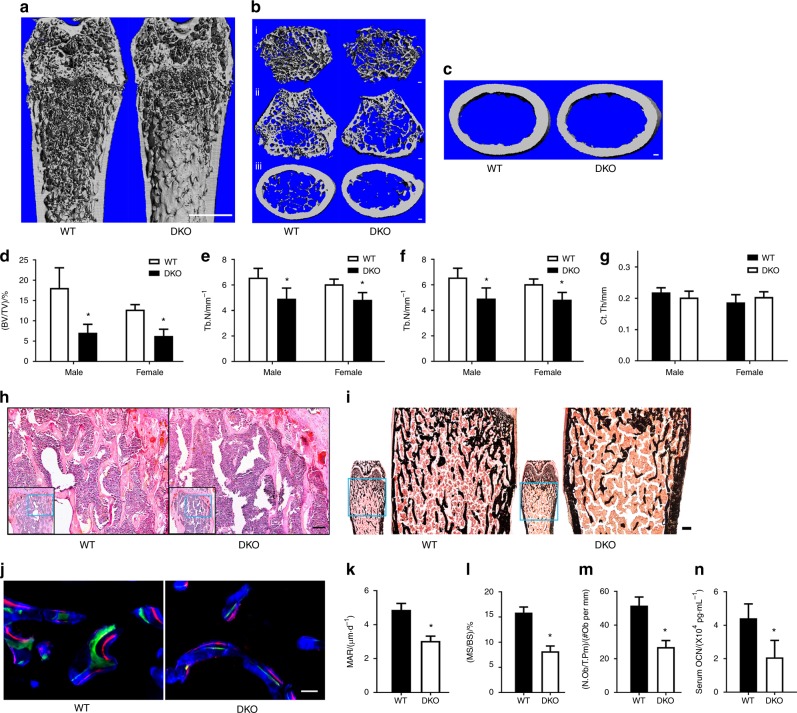
The  double knockout of *Numb* and *Numbl* causes osteopenia and inhibits bone formation in vivo. Representative three-dimensional (3D) images of femurs in 9-week-old WT or DKO mice. **a** Coronal section of distal femurs, scale bar, 1 mm. **b** 3D reconstruction of trabecular bones under femoral growth plate (i), scale bar, 100 μm; transverse slices at growth plate (ii) and at the level 300 segments away (iii), scale bar, 100 μm. **c** Transverse images of cortical bone at the level 500 segments below the growth plate, scale bar, 100 μm. Definition and description of 3D outcomes for bone micro-architecture (**d**) bone volume fraction (BV/TV). **e** Trabecular number (Tb.N). (**f**) Trabecular thickness (Tb.Th). **g** Cortical bone thickness (Ct.Th). The results are presented as the mean ± SD of at least 5 mice per group, males and females are described separately. **h** Paraffin sections of distal femurs stained with H&E. Scale bar 100 μm. **i** Frozen sections of distal femurs stained with Von Kossa (black), counterstained with Van Gieson (red). Scale bar, 200 μm. **j** Double-labelling with calcein (green) and xylenol orange (red) on trabecular bone. Bone tissues are marked by calcein blue (blue), scale bar, 50 μm. Mineral apposition rate (**k**) (MAR, *n* = 10) and mineral surface rate (**l**) (MS/BS, *n* = 10) quantification were evaluated based on double-labelling. Data shown represent the mean ± SEM, *P < 0.05. **m** Number of osteoblasts per trabecular bone perimeter (N.Ob/T.Pm, *n* = 10) quantified after toluidine blue staining. Data shown represent the mean ± SEM, *P < 0.05. **n** Concentration of OSTEOCALCIN (OCN, *n* = 12) in serum tested by ELISA assay. Data shown represent the mean ± SEM, *P < 0.05

No apparent gross abnormality of the DKO mice was observed at the age of 9 weeks (Supplementary Fig. S3b). However, a slight decrease in their body weights was noticed  (Supplementary Fig. S3c). More importantly, microcomputerized tomography revealed a conspicuous osteopenia in the femurs of DKOs (Fig. 1a, Supplementary Fig. S5 and Table 1). The trabecular bone mass was dramatically reduced (Fig. 1b–f and Supplementary Fig. S4a), while the BMD displayed no changes in these samples (Supplementary Fig. S4b). However, the cortical bone mass did not show a similar decline. No statistically significant differences were observed in the cortical thickness (Ct.Th; Fig. 1c, g) and the cortical area fraction (BA/TA; Supplementary Fig. S4c). Histologically, H&E (Fig. 1h) and Von Kossa staining (Fig. 1i) showed that the distal femurs of DKO mice did suffer a remarkable decrease in trabecular bone mass. Allowing for neither single knockout nor haploinsufficiency of the *Numb* and *Numbl* genes resulted in obvious changes in the bones (Supplementary Fig. S5a, b). The data  indicates that both *Numb* and *Numbl* deficiency in osteoblasts led to severe bone loss, especially in the trabecular bones.

**Table 1:** μCT Assessment of 9-week-old WT and DKO Mice

Parameters	Male			Female		
	WT(*n* ≥ 5)	DKO(*n* ≥ 5)	*P* value	WT(*n* ≥ 5)	DKO(*n* ≥ 5)	*P* value
(BV/TV)/%	18.10 ± 4.96	7.08 ± 2.06^*^	0.009 7	12.76 ± 1.22	6.30 ± 1.65^*^	0.000 1
Tb.Th/mm	0.046 9 ± 0.004 2	0.031 8 ± 0.001 7^*^	0.001 3	0.036 9 ± 0.001 5	0.031 9 ± 0.001 5^*^	0.002 5
Tb.N/mm	6.584 5 ± 0.718 5	4.931 9 ± 0.821 9^*^	0.030 0	6.055 1 ± 0.408 3	4.845 7 ± 0.555 9^*^	0.008 5
Tb.Sp/μm	0.155 9 ± 0.014 8	0.209 7 ± 0.036 3^*^	0.037 1	0.168 2 ± 0.011 6	0.210 9 ± 0.023 5^*^	0.012 9
BMD/(g·mm^-^^3^)	1.030 9 ± 0.115 8	0.761 0 ± 0.086 3	0.180 9	0.930 0 ± 0.033 4	0.759 1 ± 0.074 4	0.160 2
Ct.Th/mm	0.218 5 ± 0.015 0	0.202 5 ± 0.020 0	0.214 5	0.186 9 ± 0.024 7	0.204 2 ± 0.016 4	0.480 1
(BA/TA)/%	41.65 ± 3.78	36.47 ± 3.50	0.096 4	40.17 ± 0.025 0	0.386 1 ± 0.020 1	0.244 9

μCT was performed on femurs. Values represent the mean ± SD; *n* ≥ 5

*BV/TV* bone volume/total volume (bone volume fraction), *Tb.Th* trabecular thickness, *Tb.N* trabecular number, *Tb.Sp* trabecular separation, *BMD* bone mineral density, *Ct.Th* cortical bone thickness, *BA/TA* bone area/total area (bone area fraction)

*Significantly different from WT by unpaired *t* test, *P* < 0.05

### *Numb*/*Numbl* loss impaired osteoblast survival and osteogenic differentiation

Bone histomorphometry of the distal femurs in 9-week-old mice was performed in DKO mice (Table 2). Toluidine blue staining revealed a significant decline in N.Ob/T.Pm (Fig. 1m). The MAR (Fig. 1k) and the MS/BS (Fig. 1l) decreased by 37.58% and 48.49%, respectively, based on measurements from double labelling (Fig. 1j). Especially in the serum of DKO mice, the concentration of OCN dropped dramatically (Fig. 1n). These  results suggest that the double knockout of *Numb* and *Numbl* suppresses bone formation .

**Table 2:** Histomorphometric Analysis of Bone Parameters in the Femurs of 9-week-old Mice

Parameter	WT (*n* = 10)	DKO (*n* = 10)	*P* value
(N.Ob/T.Pm)/ (#Ob per mm)	51.54 ± 5.08	26.98 ± 3.82*	<0.001
MAR/(μm·d^-1^)	4.87 ± 0.38	3.041 ± 0.28*	<0.001
(MS/BS)/%	15.86 ± 1.12	8.17 ± 1.06*	<0.001
(N.Oc/T.Pm)/(#Oc per mm)	4.14±0.53	7.21±0.6^*^	<0.001
(Oc S/BS)/%	9.73±0.80	15.96±1.17*	<0.001
Oc surface/(μm per Oc)	30.09±2.36	39.54±5.77*	0.001

Histomorphometry was performed on femurs. Values represent the mean ± SD; *n* = 10

^a^Significantly different from WT by unpaired t test, *P*<0.05 N.Ob/T.Pm: osteoblasts per trabecular bone perimeter;

*N.Ob/T.Pm* osteoblasts per trabecular bone perimeter, *MAR* mineral apposition rate, *MS/BS* mineral surface rate, *N.Oc/T. Pm* osteoclast number/tissue perimeter ratio, *N.Oc/BS* osteoclast number/bone surface

*Significantly different from WT by unpaired *t* test, *P* < 0.05

**Fig. 2:**
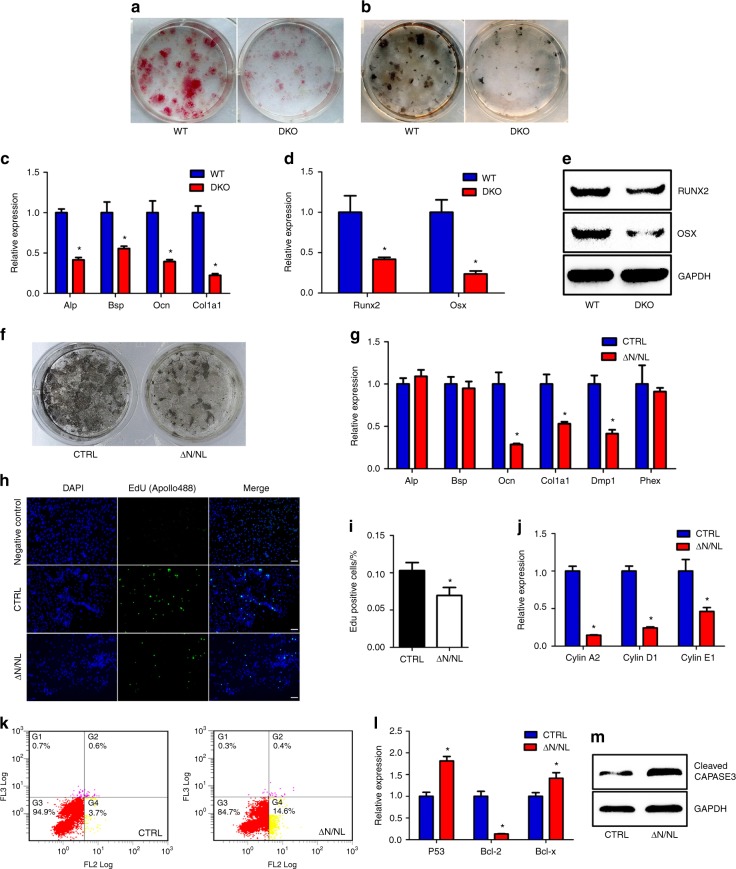
The *Numb* and *Numbl* excisions in osteoblasts inhibit proliferation and differentiation and promote apoptosis. Bone marrow stromal cells (BMSCs) were cultured in complete medium and treated with osteogenic differentiation medium on day 7. Alkaline phosphatase staining (**a**) was performed on day 7 (red), and Von Kossa staining (**b**) was performed on day 29 to mark the mineralized matrix (black). **c** The mRNA levels of osteogenic genes (*Alp*, *Bsp*, *Ocn* and *Col1a1*) and (**d**) key transcription factors (*Runx2* and *Osx)* in BMSCs cultures were analysed by RT-qPCR and normalized to β-actin. Data shown represent the mean ± SEM, *P < 0.05. **e** Western Blot for RUNX2 and OSX. Calvarial osteoblasts isolated from N/NL-floxed mice were transfected with adeno-GFP (CTRL) or adeno-Cre-GFP (ΔN/NL) viruses and cultured in osteogenic medium. GAPDH was used for normalization. **f** Von Kossa staining was performed 21 days after adenoviral infection. **g** The qRT-PCR results of bone markers (*Alp*, *Bsp*, *Ocn*, *Col1a1*, *Dmp1* and *Phex*) two days after Cre transfection. β-actin was used for normalization. Data shown represent the mean ± SEM, *P < 0.05. **h** Representative images of 5-ethynyl-2’-deoxyuridine (EdU) staining (green) on day 2 to evaluate cell proliferation; nuclei were counterstained with DAPI (blue). Scale bar, 50 μm. **i** EdU-positive cells were quantified after normalization to DAPI-positive cells. Data shown represent the mean ± SEM, *P < 0.05. **j** Cell proliferation markers (*Cyclin A2, Cyclin D1* and *Cyclin E1*) in ΔN/NL osteoblasts. β-actin was used for normalization. Data shown represent the mean ± SEM, *P < 0.05. **k** Dot plots about osteoblast apoptosis resulted from flow cytometry on day 2. Annexin V-PE was scanned in FL2, while 7-AAD was scanned in FL3. **l** qRT-PCR results for cell apoptosis markers (*P53*, *Bcl-2* and *Bcl-x*). β-actin was used for normalization. Data shown represent the mean ± SEM, * *P* < 0.05. **m** Western Blot for cleaved CASPASE-3. GAPDH was used for normalization

Primary BMSCs were isolated and  cultured   in an osteogenic medium to assess impaired  differentiation. The staining of ALP on day 7 showed that the ALP-positive colonies in the DKO groups were maintained at an extraordinarily low level (Fig. 2a). As expected, the number of mineralized colonies were also decreased dramatically on day 28 (Fig. 2b). At the transcriptional level, the expression of bone markers, including *Alp*, *Bsp*, *Ocn*, and *Col1a1*, declined sharply in the DKO groups (Fig. 2c). Most noticeably, the expression levels of key transcriptional factors, *Runx2*, and *Osx* were downregulated, as shown by the qRT-PCR (Fig. 2d) and Western Blot (Fig. 2e).

The primary osteoblasts were isolated from the N/Nl-floxed mice calvaria and gene knockout was induced by adding Adeno-Cre-GFP virus (ΔN/NL osteoblasts) to specifically evaluate the impact of *Numb/Numbl* on osteoblasts. The osteoblast-specific deletion of *Numb*/*Numbl* diminished the area of the mineralized matrix (Fig. 2f). The expression of *Ocn*, *Col1a1*, and *Dmp1* decreased, while the expression of *Alp*, *Bsp*, and *Phex* showed no obvious change (Fig. 2g). Thus, double knockout of *Numb* and *Numbl* lowered both the differentiation and function of the osteoblasts.

The 5-ethynyl-2´-deoxyuridine (EdU) assay was performed in vitro to test the cell proliferation rate in ΔN/NL osteoblasts. When normalized, the number of EdU-positive osteoblasts in the DKO group was lower than that in the WT group (Fig. 2h and i). The qRT-PCR analysis showed that the expression of critical genes involved in ΔN/NL osteoblast proliferation (*cyclin A2, cyclin D1*, and *cyclin E1*) declined at different levels (Fig. 2j). Next, the cell apoptosis rate was examined using flow cytometry after double staining the cells with Annexin V–PE (FL2) and 7-AAD (FL3). The proportion of cells in early apoptosis increased from 3.7% to 14.6% in the case of double knockout of *Numb* and *Numbl* (Fig. 2k). The expression of *P53* and *Bcl-x* increased, while the expression of *Bcl-2* was suppressed (Fig. 2l). Western Blot analysis revealed a remarkable rise in cytoplasmic-cleaved caspase-3 (Fig. 2m). These data indicate that *Numb*/*Numbl* knockout inhibited cell proliferation and promoted cell apoptosis, suppressing osteoblast survival.

### Deletion of *Numb*/*Numbl* increased PTEN rather than activating Notch in osteoblasts

**Fig. 3:**
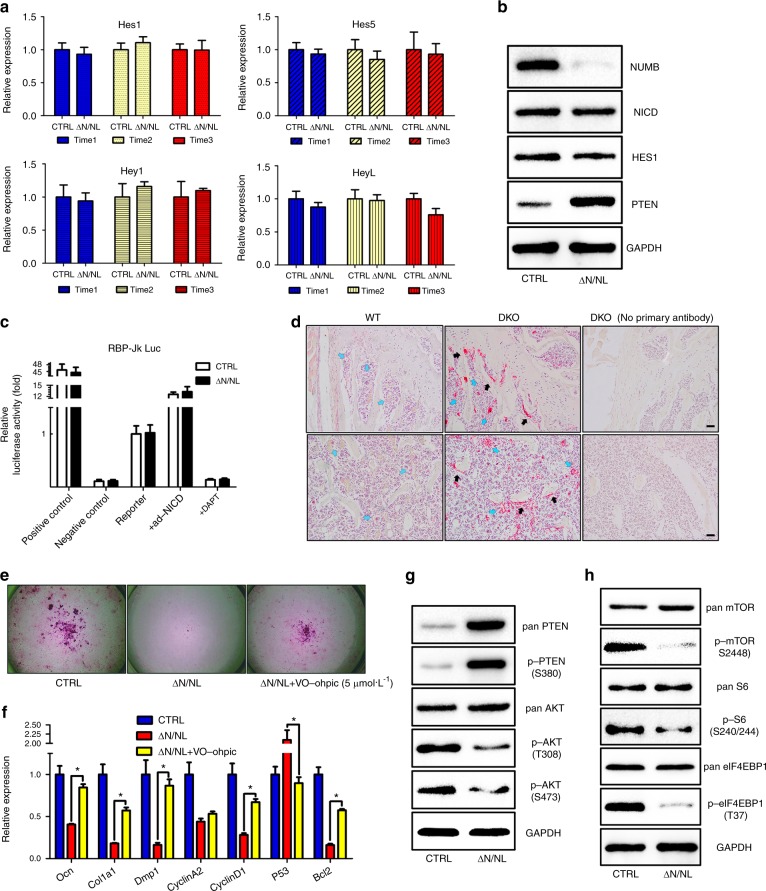
The *Numb* and *Numbl* deficiency promoted PTEN instead of Notch. **a** Triplicate qRT-PCRs to measure the expression of Notch target genes (*Hes1*, *Hes5*, *Hey1* and *HeyL*). **b** Western Blots for NUMB, NICD, HES1 and PTEN changes in ΔN/NL osteoblasts. Whole-cell lysates and RNA were extracted two days after adenovirus transduction. GAPDH was used for normalization. **c** ΔN/NL and CTRL osteoblasts were transiently transfected with an Rbp-jk reporter or positive/negative control plasmids. Extra controls of Ad-NICD (positive) and DAPT (negative) were set up to observe the efficiency of this test. Luciferase levels were normalized to Renilla luciferase and presented as fold changes to monitor Notch activation. Error bars represent the standard deviation of triplicate transfections. Data shown represent the mean ± SEM, *P < 0.05. **d** Two images of femoral paraffin sections of WT and DKO mice immunostained by anti-PTEN (red) and counterstained with haematoxylin (blue nucleus). Black and blue arrows refer to positive signals on or near the bone surface, respectively. Scale bar, 100 μm. **e** A PTEN-specific inhibitor rescued depressed osteogenesis in ΔN/NL osteoblasts. VO-Ohpic (5 µM) was incubated with ΔN/NL osteoblasts. Alizarin Red was used to mark the mineralized matrix here. **f** Bone markers (*Ocn, Col1a1 and Dmp1*); proliferation markers (*Cyclin A2, Cyclin D1*) and apoptosis markers (*P53, Bcl-2*). Data were normalized to β-actin. Data shown represent the mean ± SEM, *P < 0.05. Western blots for signal molecules in the Akt (**g**) and mTOR pathways (**h**). GAPDH was used for normalization

NUMB is conventionally considered a negative regulator of the Notch signalling pathway. Thus, the deficiency of *Numb* should increase the activity of the Notch signalling pathway^30^. However, examination of the expression of the main Notch target genes, including *Hes1*, *Hes5*, *Hey1*, and *HeyL*, did not reveal any enhanced activation of the Notch signalling activity in ΔN/NL osteoblasts (Fig. 3a). The Western Blot showed no significant changes in NICD on the hairy and enhancer of split-1 (HES1) in ΔN/NL osteoblasts (Fig. 3b). A luciferase assay was also performed to monitor the activity of Jk recombination signal-binding protein (RBP-Jk) transcription factor. However, no significant difference was found in the Notch signalling pathway between the ΔN/NL and CTRL osteoblasts (Fig. 3c). Additionally, *Numb/Numbl* knockout did not lead to a change in either the cytosolic or the nuclear NICD level (Supplementary Fig. S6).

However, PTEN, the negative regulator of phosphatidylinositide 3-kinases (PI3Ks)^31,32^, found a noticeable boost (Fig. 3b). Then, the paraffin sections of both WT and DKO mice were immunostained using an anti-PTEN antibody, and a conspicuous elevation of the expression of PTEN was found on (black arrow) or near (blue arrow) the surface of the trabecular bones (Fig. 3d). VO-Ohpic, a PTEN-specific chemical inhibitor^33^, partly rescued the osteopenic phenotype caused by a *Numb* deletion in vitro (Fig. 3e, f). In ΔN/NL osteoblasts, both pan PTEN and phosphorylated PTEN at S380 increased, while the phosphorylation of Akt at T308 and S473 decreased (Fig. 3g). Accordingly, the downregulation of the mTOR pathway (Fig. 3h) was detected. Together, these data indicate that *Numb* deficiency suppresses the Akt pathway by enhancing the cytoplasmic PTEN level, instead of the Notch level, in osteoblasts.

### Decreased ubiquitination led to accumulated PTEN in *Numb*/*Numbl*-deficient osteoblasts

**Fig. 4:**
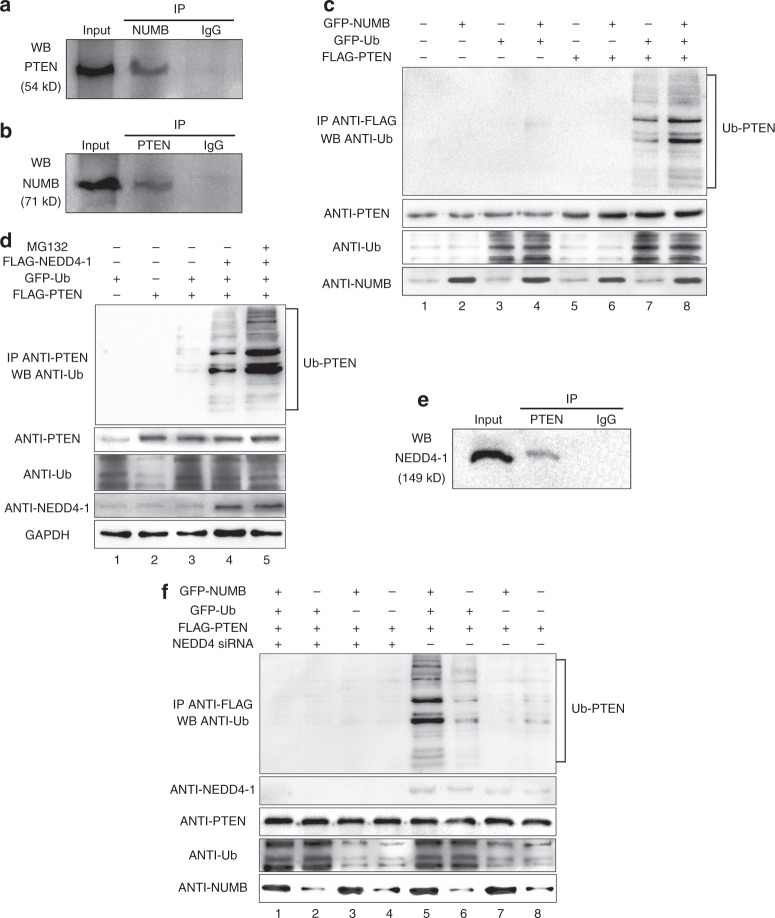
NUMB promoted ubiquitination of PTEN in osteoblasts. Co-immunoprecipitation between NUMB and PTEN in primary calvaria osteoblasts. Cell lysates were immunoprecipitated with anti-NUMB (rabbit) (**a**), anti-PTEN (rabbit) (**b**) or rabbit IgG and immunoblotted with anti-PTEN (goat) (**a**) or anti-NUMB (goat) (**b**) antibody separately. **c** NUMB facilitated the ubiquitination of PTEN. MC3T3-E1 cells were co-transfected with GFP-*m-Numb*, GFP-*m-Ubiquitin* and FLAG-*m-Pten* plasmids. Cell extracts were immunoprecipitated with anti-FLAG antibody (goat) and co-precipitated ubiquitin was identified using anti-ubiquitin (rabbit). **d** NEDD4-1 participated in the ubiquitination of PTEN in MC3T3-E1 cells. Cells were co-transfected with FLAG-m-*Nedd4-1*, GFP-m*-Ubiquitin* and FLAG-m-*Pten* plasmids. Lane 5 shows cells incubated with 10 µM MG132 for 6 h, and lanes 1–4 were incubated with DMSO. Cell extracts were immunoprecipitated with anti-PTEN antibody (goat), and co-precipitated ubiquitin was identified using anti-ubiquitin (rabbit). **e** NUMB co-immunoprecipitated with NEDD4-1 in primary calvaria osteoblasts. Cell lysates were immunoprecipitated with anti-NUMB (goat) or goat IgG and immunoblotted with anti-NUMB (rabbit) antibody. **f** The positive regulation of NUMB on the ubiquitination of PTEN could not be achieved without the attendance of NEDD4-1. MC3T3-E1 cells were co-transfected with plasmids encoding GFP-m-*Numb*, GFP-m-*Ubiquitin*, FLAG-m-*Pten* and si-*Nedd4*. Two days after transfection the cells were lysed. PTEN was pulled down with anti-FLAG agarose beads and subjected to immunoblotting with anti-ubiquitin to detect ubiquitinated PTEN (Ub-PTEN)

NUMB participates in the process of ubiquitin degradation by binding with ubiquitin ligase ITCH to cause the breakdown of NOTCH or GLI1 and to inhibit their function^20,34^. Similarly, ubiquitin-mediated proteasomal degradation of PTEN is a critical mechanism for influencing PTEN stability^35^. Therefore, NUMB might regulate the ubiquitination of PTEN. Kim et al. demonstrated that NUMB and PTEN were able to form a complex in prostate cancer cells^36^. Therefore, the possibility of physical interaction between NUMB and PTEN in osteoblasts was explored here. NUMB was co-immunoprecipitated with PTEN in primary calvaria osteoblasts to test this hypothesis. Bands of both groups were detected at corresponding sizes, which proved the endogenous interaction between NUMB and PTEN in osteoblasts (Fig. 4a, b). An ubiquitination assay was then performed, and *m-Ubiquitin*, *m-Pten*, and *m-Numb* plasmids were transfected into MC3T3-E1 cells. *m-Numb* transfection dramatically enhanced the formation of ubiquitinated PTEN (Ub-PTEN) (Fig. 4c, lane 7 versus lane 8). These results indicate that NUMB might promote the ubiquitination of PTEN in osteoblasts.

The ubiquitination of a protein needs its corresponding E3 ligase, and NEDD4–1 is a negative regulator of PTEN via ubiquitination^35^. Structurally, both ITCH and NEDD4–1 belong to the NEDD4 family whose WW domains are the main mediators in interactions with substrates and adaptors^37^. The phosphotyrosine-binding (PTB) domain of NUMB binds with the WW1/2 domain of ITCH to regulate NOTCH ubiquitination^34^, leading to the assumption that NUMB could interact with NEDD4-1 to influence its regulation of PTEN in several potential ways. Therefore, sequence alignment was performed in this study to identify regions of similarity between the human ITCH and the human NEDD4 domains (Supplementary Fig. S7a, b). The functional WW1 (Supplementary Fig. S7a) and WW2 (Supplementary Fig. S7b) domains in ITCH were similar to all four NEDD4 WW domains. Moreover, NUMB in humans and mice shared exactly the same sequences in the PTB domain (Supplementary Fig. S7c). However, ITCH shared 99.25% identity in the WW domains (Supplementary Fig. S7d). Despite the lack of the WW3 domain, mouse NEDD4 maintained a high consistency of sequence with the remaining three human domains. Notably, there was a more than 94.12% and 88.24% similarity between the WW1/2 domains of NEDD4 in humans and mice, respectively (Supplementary Fig. S7e). Hence, the aforementioned bioinformatics analyses hinted at the possibility that NUMB might be involved in the regulation of PTEN by NEDD4. At the molecular level, transfection of the *m-Nedd4-1* plasmids remarkably boosted the production of Ub-PTEN (Fig. 4d, lane 3 vs. lane 4) in MC3T3-E1 cells. Moreover, the administration of MG132, a classical chemical proteasome inhibitor, markedly promoted this process (Fig. 4d, lane 5). Next, the co-immunoprecipitation assay was performed to verify the physical interaction between NUMB and NEDD4-1 (Fig. 4e). The abating of the expression of NEDD4-1 via the introduction of *Nedd4-1* siRNA into the ubiquitination assay suppressed the overproduction of Ub-PTEN promoted by *m-Numb* transfection (Fig. 4f, lane 1 vs. lane 5). These results suggest that NUMB deletion enhanced PTEN stability by suppressing NEDD4-1-dependent ubiquitin-mediated proteasomal degradation of PTEN.

### Dislodging *Numb*/*Numbl* in osteoblasts mediated the activation of osteoclastogenesis

**Fig. 5:**
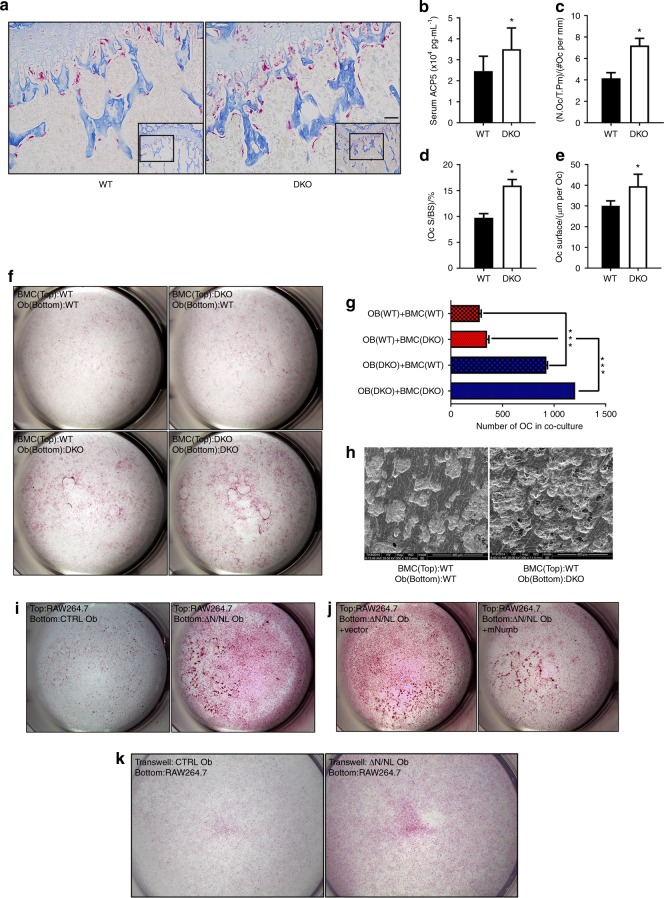
The knockout of *Numb* and *Numbl* in osteoblasts boosted osteoclastogenesis. **a** TRAP staining on paraffin sections of WT and DKO femurs to show osteoclasts on the bone surface. Scale bar, 50 μm. **b** The concentration of tartrate-resistant acid phosphatase 5 (ACP5, *n* = 12) in the serum of WT or DKO mice. Data represent the mean ± SEM, *P < 0.05. Histomorphometry based on TRAP staining: (**c**) the number of osteoclasts per trabecular bone perimeter (N.Oc/T.Pm, *n* = 10); **d** the osteoclast surface rate (Oc.S/BS, *n* = 10); (**e**) the average osteoclast surface (Oc surface, *n* = 10). Data shown represent the mean ± SEM, *P < 0.05. **f** Co-culture of WT or DKO calvaria (bottom) osteoblasts with BMC (top) from WT or DKO mice in 48-well plates, TRAP-stained and haematoxylin counterstained. **g** The number of osteoclasts were counted and are shown as the mean ± SEM, *P < 0.05. **h** Representative images of bone resorption pits from a scanning electron microscope (SEM). Scale bar, 400 μm. **i** RAW264.7 cells were co-cultured on the surface of ΔN/NL or CTRL osteoblasts in 48-well plates and were TRAP-stained. **j** *Numb* overexpression in ΔN/NL osteoblasts enhanced osteoclastogenesis. ΔN/NL osteoblasts were transfected with a GFP-m-*Numb* plasmid. Two days after transfection, RAW264.7 cells were seeded on the surface of osteoblasts, and the wells were stained with TRAP 10 days later. **k** Transwell cultures of ΔN/NL or CTRL osteoblasts on small transwell inserts with 0.4 μm pores; RAW264.7 cells were co-cultured in lower 24-well plates, and TRAP staining was performed 10 days later

TRAP staining was carried out to explore whether osteoclasts contributed to osteopenia, and a significant increase was found in the number of osteoclasts on the trabecular bone in the DKO mice (Fig. 5a). Increases in the osteoclast number/tissue perimeter ratio (N.Oc/T. Pm; Fig. 5c), osteoclast number/bone surface (N.Oc/BS; Fig. 5d), and osteoclast surface (Fig. 5e) were detected using histomorphometry (Table 2). Furthermore, tartrate-resistant ACP5 in the serum of the DKO mice was noticeably enhanced (Fig. 5b). These data provided evidence of an upgrade in the bone resorption activity in DKO mice.

An osteoblast-bone marrow cell (BMC) co-culture assay was performed to examine the effect exerted by the loss of *Numb* and *Numbl* in osteoblasts on osteoclastogenesis (Fig. 5f). BMCs that had been seeded on DKO osteoblasts produced far more osteoclasts than those seeded on WT osteoblasts (Fig. 5f, g). A greater resorption area resulting from osteoclasts was found in the DKO groups (Fig. 5h). When the RAW264.7 macrophage cell line instead of BMCs was co-cultured, *Numb*-deficient osteoblasts stimulated more TRAP-positive cells (Fig. 5i). Moreover, the *m-Numb* plasmid transfection remarkably attenuated this osteoclast-induced effect of ΔN/NL osteoblasts (Fig. 5j). Furthermore, the transwell assay was performed by seeding ΔN/NL or CTRL osteoblasts in the upper wells with 0.4-μm pore size while co-culturing RAW264.7 cells on the lower 24-well plates. The ΔN/NL osteoblasts were found to stimulate more TRAP-positive multi-nucleated RAW264.7 cells (Fig. 5k and Supplementary Fig. S8). Given that no cell–cell direct contact or cell migration existed between these two chambers, the results demonstrated that ΔN/NL osteoblasts promoted osteoclastogenesis via certain secretory and soluble factors.

### Enhanced osteoclastogenesis due to the elevation of RANKL/OPG ratio

**Fig. 6:**
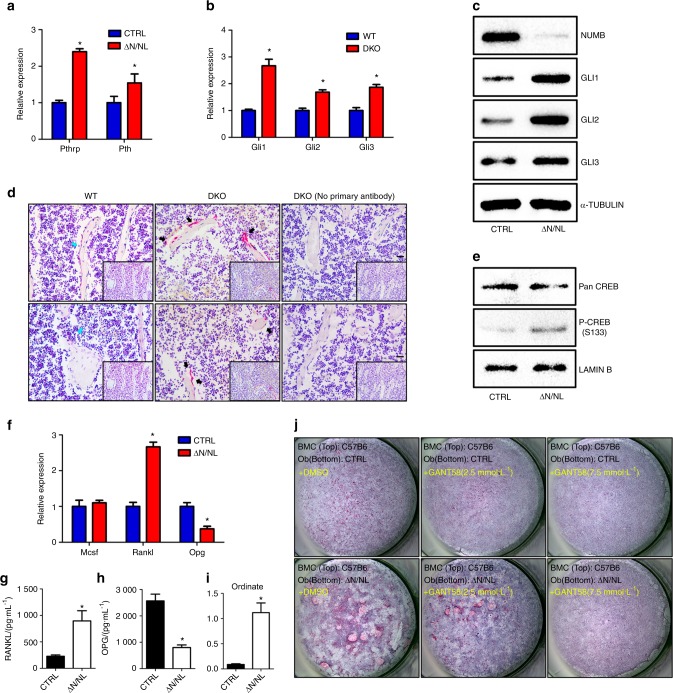
The *Numb*-abated osteoblasts enhanced RANKL/OPG via the Hedgehog pathway. **a** qRT-PCR results of *Pthrp* and *Pth* transcriptional changes. qRT-PCRs were normalized to β-actin, and the data represent the mean ± SEM, *P < 0.05. **b** The mRNA and (**c**) protein levels of Gli1-3 in ΔN/NL osteoblasts were analysed separately by qRT-PCR and Western Blot. qRT-PCRs were normalized to β-actin, and Western Blot data were normalized to α-TUBLIN. **d** Two representative images of paraffin sections of WT and DKO femurs immunostained with anti-GLI1 (red, black arrow) and haematoxylin counterstained. Blue arrows indicate the moderate expression of NUMB in the WT mice. Scale bar 20 μm. **e** The increased formation of phosphorylated CREB (p-CREB) in the nuclei of ΔN/NL osteoblasts was detected by Western Blot and normalized by LAMIN B. **f** *Mcsf, Rankl* and *Opg* expression in ΔN/NL osteoblasts. qRT-PCRs were normalized to β-actin. Data shown represent the mean ± SEM, *P < 0.05. RANKL (**g**) and OPG (**h**) levels in conditional medium collected from ΔN/NL and CTRL osteoblast culture and defined by ELISA assay. The RANKL/OPG ratio is remarkably increased (**i**) *n* = 12. The data shown represent the mean ± SEM, *P < 0.05. GLI1-specific inhibition attenuated the osteoclast-inducing effect of the ΔN/NL osteoblasts (**j**). BMCs from C57B6 mice were seeded on ΔN/NL or CTRL osteoblasts after a 12 h incubation with GANT58 (2.5  or 7.5 μmol·L^-1^) or DMSO. Wells were TRAP-stained and haematoxylin counterstained 12 days later

NUMB has been found to target the Hh transcriptional factor GLI1 and inhibit the Hh signal through ITCH-dependent ubiquitination mechanisms^20,21^. The expression of *Gli1*, *Gli2*, and *Gli3* increased in both the total RNA extracted from the long bones of DKO mice (Fig. 6b) and the total protein extracted from ΔN/NL osteoblasts (Fig. 6c). Furthermore, excessive expression of GLI1 was found on the surface of trabecular bones in DKO mice using anti-GLI1 (Fig. 6d, black arrows). Thus, *Numb* and *Numbl* knockout activated the Hh pathway in osteoblasts.

The abnormally high-level Hh pathway in the osteoblasts led to osteopenia in adult mice. The elevated expression of parathyroid hormone-related protein (PTHrP) caused by hyperactivity of Hh was responsible for the increase in bone resorption^10^. Consistent with these results, a high expression of *Pthrp* and *Pth* at the transcriptional level was found in this study in ΔN/NL osteoblasts (Fig. 6a). Additionally, a Western Blot analysis detected the enhanced phosphorylation of CREB protein, the transcription factor targeted by protein kinase A (Fig. 6e). Furthermore, qRT-PCR demonstrated an increased expression of *Rankl* and a decline in the expression of *Opg*. However, the expression of *Mcsf* did not change significantly (Fig. 6f). At the same time, ELISA data revealed a significant increase in RANKL (Fig. 6g) and a decrease in OPG (Fig. 6h) in the conditional media in the wells of ΔN/NL osteoblasts. The RANKL/OPG ratio in the ΔN/NL group was 12.48 times higher than that in the CTRL group (Fig. 6i). Finally, GANT58, the GLI1-specific chemical inhibitor, antagonized the osteoclast-inducing effect of ΔN/NL osteoblasts in a dose-dependent manner (Fig. 6j). Together, these results demonstrated that the deletion of *Numb* and *Numbl* activated the Hh pathway and boosted osteoclastogenesis by upregulating the RANKL/OPG ratio.

## Discussion

In this study, the function of *Numb* and its homologue *Numbl* in bone was systematically investigated. Data from the experiments suggest that *Numb* and *Numbl* in osteoblasts maintain the survival of osteoblasts by sustaining the Akt level via downregulating PTEN.  In addition, *Numb* and *Numbl* suppressed bone resorption by inhibiting the Hh signal. Thus, it was concluded that *Numb* and *Numbl* maintain bone mass.

As NUMB is thought to be an antagonist of Notch  and  in inducible Col1a1-3.2-Cre^ERT2^ mice, the conditional overexpression of Notch in mature osteoblasts and osteocytes triggers remarkable bone formation in adult bones^38^, *Numb* deficiency was hypothisized to stimulate the Notch signaling and  cause a high bone mass. However, Col2.3-Cre; N/NL-floxed mice was observed an unexpected osteopenic phenotype (Fig. 1). In this study, Notch was not activated in osteoblasts (Fig. 3a–c). Originally, NUMB was recognized as a cell fate determinant. In *Drosophila*, d-numb was asymmetrically located in one daughter cell in which the Notch signal was suppressed^30^. In mammalian cells, a high level of Notch reduced the levels of both *Numb* and *Numbl*, accompanied by a reciprocal negative regulatory interaction between Notch and NUMB^39^. Mechanically, NUMB controls endocytosis and degradation of the Notch receptor or its intracellular domain by ubiquitination^34,40,41^. However, a growing body of evidence reveals mammalian NUMB to be more than just a Notch antagonist^14^. NUMB is a versatile endocytic protein. Apart from Notch, NUMB was also reported to promote endocytic degradation of ERBB2 or integrin in cardiomyocytes to affect cell migration^42,43^. NUMB is also a multifaceted ubiquitination adaptor. It regulates signalling pathways via ubiquitination of specific substrates. For instance, NUMB suppresses the degradation of P53 by ubiquitination via the formation of a NUMB–HDM2–P53 tri-complex to inhibit tumourigenesis^18,44^. Moreover, NUMB can bind with the WW2 domain of ITCH to release ITCH from its self-repressive form, leading to the degradation by ubiquitination of the Hh pathway effector GLI1^20,21^. Thus, mammalian NUMB is more than a Notch antagonist and harbours functional diversity in different cell types and distinct developmental stages.

In osteoblasts, a mechanism was discovered by which NUMB influenced NEDD4-1-mediated ubiquitination of PTEN. Ubiquitination is a posttranslational modification and E3 ubiquitin ligases determine the specificity of the substrates, leading to the degradation of receptor tyrosine kinases of the signalling molecules and of the transcription factors in specific pathways^45^. Generally, mammalian E3 ligase can be classified into two groups: the really interesting new gene (RING) finger and the homologous to E6-AP COOH terminus (HECT) domain. Both NEDD4-1 and ITCH belong to the HECT domain-containing NEDD4 family^37^. NUMB regulates ubiquitination through its evolutionarily conserved PTB domain and this regulation needs the mediation of ITCH^21,34^. Structurally, NEDD4-1 and ITCH share high similarity in WW1/2 domains that interact with the PTB domain of NUMB (Fig. 4c, d)^34^. The co-immunoprecipitation assays confirmed that NUMB, PTEN, and NEDD4-1 might work by physiologically forming a complex in osteoblasts (Fig. 4). In mammalian cells, NEDD4-1 ubiquitinates PTEN and results in ubiquitin-mediated proteasomal degradation^35^. Rak, a tyrosine kinase in the Src family, is a negative regulator in this process. Physiologically, it represses PTEN ubiquitination by adjusting the binding of PTEN to NEDD4-1. Moreover, it phosphorylates PTEN on Tyr336 to optimize the stability of PTEN. Furthermore, the knockdown of Rak markedly promotes the growth of tumour cells^46^. In this study, NUMB, in contrast to the negative regulator Rak, is a positive adaptor of PTEN ubiquitination in osteoblasts. Recently, Shao et al. found that such regulation existed in multiple human cell lines. NUMB influences the interaction between PTEN and NEDD4-1 and the nuclear localization of PTEN. The regulation of PTEN by ubiquitination participates in oncogenesis^47^. On the basis of investigations in vivo, the positive effect of NUMB on PTEN was proven to be inevitable for normal cellular metabolism. Further, NUMB is a crucial adaptor protein that promotes the degradation of the Akt, Hh, P53, Notch, and other known/unknown pathways by ubiquitination. However, it is unclear when and where it regulates Notch and when and where it controls Akt. Moreover, it is unclear whether there are any switch points controlled by different cell contexts. Further studies are needed to address these important issues.

The ubiquitination-proteasomal degradation system is crucial to regulating bone cell functions^48^. In this study, NUMB physiologically promoted the Akt signal by negatively controlling the cytosolic level of PTEN by ubiquitination (Figs. 3 and 4). Akt controls osteoblast–osteoclast coordination. Akt1^−/−^ mice suffer from similar osteopenia. Akt1-deficient osteoblasts are rendered susceptible to apoptosis. Their differentiation and function are depressed and their RANKL secretion is inhibited^49^. Osteoblast-specific abating of the expression of PTEN dramatically activates the Akt pathway. Enhanced proliferation and reduced apoptosis account for the prolonged lifespan of osteoblasts and the hyperactivity of bone formation in PTEN-knockout mice^50^. Akt controls many downstream signals including mTOR^51^. The results of this study indicated that in the context of dual silencing of *Numb*/*Numbl*, the Akt–mTOR pathway was obviously inhibited. The mTOR signal is involved in skeletal development via controlling energetics and via protein synthesis in the osteoblastic lineage, where it promotes differentiation^52–55^. In mesenchymal stem cells (MSCs), mTOR activation by the insulin-like growth factor 1 or by deletion of the peroxisome proliferator-activated receptor gamma induces osteoblast differentiation to maintain the adult bone mass^25,56^. In osteoblasts, rapamycin suppresses Runx2 and Osx to inhibit differentiation and decreases cyclin A and D1 to inhibit proliferation^26^. Moreover, in MC3T3-E1 cells, a decreased Akt-mTOR level caused by high glucose leads to a high level of apoptosis and low proliferation. In this context, the mTOR-specific chemical activator can ameliorate dexamethasone-induced apoptosis^57,58^. This study revealed in bone remodelling that mTOR in osteoblasts was still a prosurvival signal that made differentiation and proliferation possible while simultaneously resisting apoptosis.

NUMB is a tumour suppressor because of not only the protective function that it exercises towards P53 but also its inhibitory regulation on the Hh signal through the ubiquitination of GLI1^18,20,21^. However, this study demonstrated a similar Hh-inhibitory effect of NUMB in osteoblasts. The results highlighted the key role of GLI1 in the mediation between *Numb*-deficient osteoblasts and increased bone resorption. In bones, GLI1 is found in MSCs with a high potential for osteogenic differentiation^59^. GLI1 is involved in the Hh-mediated specification of osteoblasts and the stimulated early differentiation in endochondral ossification^60^, whereas in postnatal bone modelling/remodelling, a low bone mass phenotype was observed in mice subjected to *Gli1* global haploinsufficiency^61^. Mak et al. found that the physiological activity of Hh was progressively reduced as osteoblast matured. The use of an osteoblast-specific Osteocalcin-Cre line to conditionally stimulate high levels of Hh signal showed that excessive bone resorption overwhelmed a limited increase in bone formation leading to a severe osteopenia phenotype^10^. Similarly, the loss of *Numb* activated the Hh signal by promoting the expression of GLIs. Elevation of PTHrP was found to enhance the phosphorylation of CREB and increase the RANKL/OPG ratios (Fig. 6e–i)^62^. Therefore, apart from the depressed osteoblast survival rate caused by increased PTEN, GLI1-meditated activation of Hh in *Numb*-abated osteoblasts contributed to osteopenia by influencing secretory processes such as RANKL and OPG.

**Fig. 7:**
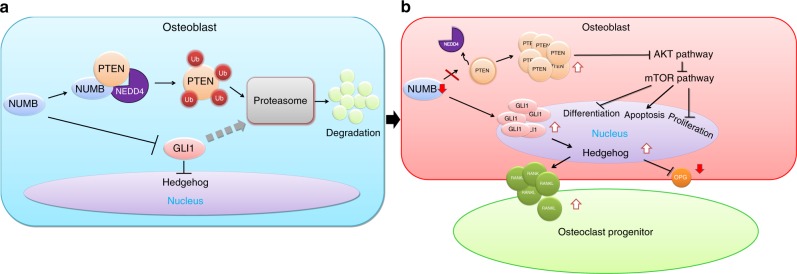
*Numb* and *Numbl*in osteoblasts maintain the physiological bone mass. **a** When expressed normally in osteoblasts, the NUMB-NEDD4-PTEN tri-complexes produce, leading to ubiquitin-mediated proteasomal degradation of PTEN. At the same time, GLI1 tends to be degraded in proteasomes. Akt and Hedgehog signals are moderate in osteoblasts. **b** When *Numb* is suppressed, PTEN and GLI1 accumulate in the cytoplasm where they inhibit Akt to suppress bone formation and activate Hedgehog to enhance bone resorption, respectively

In summary, this study emphasizes the pivotal role of NUMB in bone homeostasis. Bone mass hinges on a delicate osteoblast–osteoclast balance struck by NUMB and NUMBL via Akt and Hh signals (Fig. 7a, b). Theoretically speaking, NUMB may be a crucial ubiquitination regulator that coordinates signalling pathways in certain cell contexts. Practically, NUMB is a potential therapeutic target for rebalancing bone loss and restoring bone strength in patients with OP.

## Materials and Methods

### Mice

*Numb*^*flox/flox*^*/Numbl*^*flox/flox*^ (N/Nl-floxed) mice were purchased from Jackson Laboratory (Stock Number: 005384). Col1a1-2.3-Cre (2.3-Cre) mice were kindly provided by David Rowe. Hemizygous 2.3-Cre mice were crossed with N/Nl-floxed mice to generate a 2.3-Cre; N/Nl-floxed allele. The offspring were viable and fertile. The pups were weaned and genotyped 21 days after birth. Both 2.3-Cre and N/Nl-floxed mice genotyping strategies exactly followed the standard protocols from the JAX Mice Database. All animals were maintained in the core facility of gene-engineered mice at the State Key Laboratory of Biotherapy. Mouse experiments were performed in accordance with institutional guidelines. Corresponding protocols were reviewed and approved by the Research Ethics Committee, West China Hospital of Stomatology, Sichuan University, Chengdu, China.

### Micro-computed tomography analysis

Femurs were isolated from 9-week-old mice, fixed in 10% formalin and transferred to 70% ethanol. The VIVA 40CT 64GB (Scanco Medical AG, Bassersdorf, Switzerland) System was used to scan 500 layers proximal to the tibial plateau. The microstructural properties of the trabecular bone, including BV/TV, Tb.Th, Tb.N, Tb.Sp, and bone mineral density (BMD), were calculated based on the 100 layers next to the growth plate. BA/TA was evaluated at 500 layers. A Gaussian filter (σ, 0.8; support, 1) was applied to the scans with a voxel of 10 μm. Corresponding parameters were set up as follows: X-ray tube potential, 55 kVp; X-ray intensity, 145 μA; integration time, 200 ms; and threshold, 220 mg·cm^-^^3^.

### Histomorphometric analysis

Calcein (3 mg·kg^-1^; Sigma) and xylenol orange (90 mg·kg^-1^; Sigma) were separately injected into 9-week-old mice 10 and 2 days before sacrificing. Femurs or tibiae were fixed in 4% paraformaldehyde (PFA) for 24 h. For frozen sections, undecalcified bones were dehydrated with 30% sucrose solution for 24 h, embedded into the optimum cutting temperature compound (Sakura), cut at a thickness of 5–7 μm using a Leica CM1850 UV cryostat, and transferred with a tape system. After being counterstained with 1% calcein blue (Sigma), some sections were photographed under a fluorescence microscope (Olympus IX71) to determine the mineral apposition rate (MAR) and mineral surface rate (MS/BS), while some were selected for Von Kossa/Van Geison staining. For paraffin sections, femurs were decalcified in 15% ethylenediamine tetraacetic acid for approximately 1 month, dehydrated in graded ethanol, infiltrated in xylene, and carefully embedded in paraffin. Consecutive sections of approximately 5 μm were stained with haematoxylin and eosin (H&E) and photographed (Olympus BX53). Toluidine blue staining was administered to quantify the number of osteoblasts per trabecular bone perimeter (N.Ob/T.Pm), and tartrate-resistant acid phosphatase (TRAP) staining was performed to determine the bone resorption-related index. This included the N.Oc/T.Pm, Oc.S/BS and the osteoclast surface.

### Primary cell culture

Bone marrow stromal cells were flushed from the femurs and tibiae of 9-week-old WT and DKO littermates using a 25-gauge needle. They were then cultured in modified alpha-MEM (HyClone) supplemented with 10% foetal calf serum (HyClone), and 1% penicillin/streptomycin (HyClone). Osteogenic differentiation was induced on day 7 in a medium containing 10 nmol·L^-1^ dexamethasone (Sigma), 10 mmol·L^-1^ β-glycerol phosphate (Sigma), and 50 μg·mL^-1^ ascorbic acid (Sigma). The cultures were terminated on day 28. Accordingly, Burstone’s alkaline phosphatase (ALP) staining was performed on day 7 to observe positive colonies, and Von Kossa staining was performed on day 28 to check mineralization.

Primary osteoblasts were digested from 2-month-old N/Nl-floxed mice calvarias. They were cultured in a complete medium after digestion with 0.2% collagenase II (Sigma) and 0.25% trypsin (HyClone). Induced cell differentiation and maturation were performed as discussed previously. Gene knockout was stimulated by adding adeno-GFP (control) or adeno-cre-GFP (ΔN/NL) virus (Shanghai Hanhbio) and refreshing the medium 2 h later. Then, 5 μmol·L^-1^ VO-Ohpic (MedChem Express) was used to determine the influence of PTEN on DKO osteoblasts. Either Von Kossa or Alizarin red staining was carried out to check mineralization. Osteoblasts were incubated with a medium containing 50 μmol·L^-1^ EdU (Guangzhou Ribobio) for 2 h to evaluate cell proliferation. Then, Apollo488 staining was performed according to the protocol provided, and EdU-positive cells were counted using a fluorescence microscope (Olympus) after being counterstained with 4’,6-diamidino-2-phenylindole. Annexin V–PE/7–AAD Detection (Keygen) was administered according to the protocol provided to evaluate cell apoptosis. Flow cytometry (Beckman Cytomics TM FC 500) was then used to analyse the proportion of cells at early and late stages of apoptosis.

### Luciferase assay

The Cignal RBP-Jk Reporter (luc) Kit (Qiagen), including an RBP-Jk reporter, with positive and negative control plasmids, was used. In 96-well plates, these plasmids were transfected to ΔN/NL osteoblasts using Lipofectamine 3000 (Invitrogen). Adeno-NICD-GFP (Shanghai Hanhbio) and a γ-secretase-specific chemical inhibitor DAPT (MedChem Express) were used as extra controls at the Notch level. After 48 h, the medium was removed and the cells were washed gently with phosphate-buffered saline (PBS) buffer. Then, 20 μL passive lysis buffer was added to the dual-luciferase reporter assay system (Promega), and the plates were shaken at room temperature for 15 min. The luciferase assay reagent II was transferred into the wells, and firefly luminescence was measured using a luminometer (Varioskan Flash; Thermo Scientific). Then, a 100 μL Stop&Glo reagent was added, and the Renilla luciferase activity was immediately recorded. All transfection procedures were carried out in triplicate.

### Immunohistochemistry and immunocytochemistry

Tissue sections or cells were washed using PBS with 0.2% Tween-20 (Sigma) or 0.5% Triton-X100 (Sigma) and stained based on the standard protocol for the Vectastain ABC-AP Kit (Vector Laboratories) or the rabbit HRP-DAB Kit (R&D System). Haematoxylin was used for counterstaining, and a neutral balsam was used as a mounting medium. A microscope imaging system (Olympus BX53) was used for imaging. The primary antibodies involved were as follows: anti-NUMB (Abcam, ab14140); anti-PTEN (Cell Signalling Technology, 9188); and anti-GLI1 (Santa Cruz Biotechnology, sc-20687).

### Immunofluorescent staining

Cells fixed in 4% PFA were washed using PBS supplemented with 0.2% Tween-20 (Sigma) or 0.5% Triton-X100 (Sigma) and incubated overnight with anti-NICD (Cell Signalling Technology, #4147). Goat anti-rabbit IgG H&L (Alexa Fluor® 594, ab150080) was used to mark NICD in red. Pictures were taken under a fluorescence microscope (Olympus IX71).

### RNA extraction and quantitative reverse transcription-polymerase chain reaction analysis

Total RNA was extracted from long bones or cell cultures. In the former case, the attached muscles were cleaned off and the bone marrow was flushed away. After freezing in liquid nitrogen, bone tissues were ground into powder and homogenized with TRIzol (Invitrogen) and then the suspension was centrifuged. The next steps were carried out following the protocol provided by TRIzol. The concentration, purity, and integrity of the RNA samples were identified using the NanoDrop ND-2000 (Thermo Scientific) and by electrophoresis using denaturing formaldehyde gels. cDNA was synthesized using the QuantiTect Reverse Transcription kit (Qiagen), and a quantitative reverse transcription-polymerase chain reaction (qRT-PCR) was performed in a CFX96 Real-Time System (Bio-Rad) using the QuantiTect SYBR Green PCR Kit (Qiagen). β-actin was chosen as an internal control, and relative quantification was used for calculating the results (Ct). Similar procedures were carried out for RNA extraction and qRT-PCR of the cell cultures. The sequences of the qRT-PCR primers are listed in the supplementary information (Table 3).

**Table 3:** Primer sequences involved in qRT-PCR amplification

Gene	Forward (5′–3′)	Reverse (5′–3′)
*Numb*	GGCCTTCACTGCTTTCTTTC	CACATCTGTGAAGATGCCGT
*Numbl*	GTGACTCCGCATTCCTTCTC	TCCTGGCCCTCAAGGACT
*Alp*	CCAGCAGGTTTCTCTCTTGG	CTGGGAGTCTCATCCTGAGC
*Bsp*	CAGGGAGGCAGTGACTCTTC	AGTGTGGAAAGTGTGGCGTT
*Ocn*	CCTTCATGTCCAAGCAGGA	TGCTGTGACATCCATACTTGC
*Col1a1*	GCCTTGGAGGAAACTTTGCTT	GCACGGAAACTCCAGCTGAT
*Dmp1*	AGTGAGGAGGACAGCCTGAA	GAGGCTCTCGTTGGACTCAC
*Phex*	CGCCTGACAAACTTTTGAGACC	TGCTCCCTGTTTCTGCTTCC
*Runx2*	TCTGGCCTTCCTCTCTCAG	GGATGAAATGCTTGGGAAC
*Osx*	ATGGCGTCCTCTCTGCTTG	TGAAAGGTCAGCGTATGGCTT
*Cyclin A2*	GCCTTCACCATTCATGTGGAT	TTGCTGCGGGTAAAGAGACAG
*Cyclin D1*	GCGTACCCTGACACCAATCTC	CTCCTCTTCGCACTTCTGCTC
*Cyclin E1*	GTGGCTCCGACCTTTCAGTC	CACAGTCTTGTCAATCTTGGCA
*P53*	AATGTCTCCTGGCTCAGAGG	CTAGCATTCAGGCCCTCATC
*Bcl-2*	ATGCCTTTGTGGAACTATATGGC	GGTATGCACCCAGAGTGATGC
*Bcl-x*	GACAAGGAGATGCAGGTATTGG	TCCCGTAGAGATCCACAAAAGT
*Hes1*	GTCACCTCGTTCATGCACTC	TCTGGAAATGACTGTGAAGCA
*Hes5*	GTAGTCCTGGTGCAGGCTCT	AACTCCAAGCTGGAGAAGGC
*Hey1*	TCCGATAGTCCATAGCCAGG	TTGCAGATGACTGTGGATCA
*HeyL*	CAGCCCTTCGCAGATGCAA	CCAATCGTCGCAATTCAGAAAG
*Gli1*	CCAAGCCAACTTTATGTCAGGG	AGCCCGCTTCTTTGTTAATTTGA
*Gli2*	CAACGCCTACTCTCCCAGAC	GAGCCTTGATGTACTGTACCAC
*Gli3*	CACAGCTCTACGGCGACTG	CGGCCTTCTCGCTGACATC
*Pthrp*	CATCAGCTACTGCATGACAAGG	GGTGGTTTTTGGTGTTGGGAG
*Pth*	TGCAAACACCGTGGCTAAAGT	CCAGGTTGTGCATAAGCTGTAT
*Mcsf*	CTGGAAGGAGGATCAGCAAG	ATGTCTGAGGGTTTCGATGG
*Rankl*	CAGCATCGCTCTGTTCCTGTA	CTGCGTTTTCATGGAGTCTCA
*Opg*	ACCCAGAAACTGGTCATCAGC	CTGCAATACACACACTCATCACT
*β-actin*	AGATGTGGATCAGCAAGCAG	GCGCAAGTTAGGTTTTGTCA

### Co-culture and Transwell assay

On day 0, 3 × 10^4^ calvaria osteoblasts were placed in 400 μL complete medium plus 10 ng·mL^-1^ cholecalciferol (Sigma) and 10 nmol·L^-1^ dexamethasone (Sigma) in a 48-well plate. On day 2, 3 × 10^5^ freshly isolated bone marrow cells (BMCs) were gently added to the surface of the osteoblast layer. On day 5 and 9, 50% of the medium was carefully renewed without irritation to the osteoblast layer. On day 10, all the cells were fixed with 4% PFA, and TRAP staining was carried out. Osteoclasts were counted and photographed. Similar procedures were carried out where 3 × 10^5^ RAW264.7 cells were seeded on osteoblasts. For the transwell assay, osteoblasts were cultured on transwell inserts (pore size: 0.4 μm, Corning), and RAW264.7 cells were seeded on the lower compartment of a 24-well plate. After 12 days the RAW264.7 cells were fixed and stained with TRAP. In the rescue assay, concentrations of GANT58 (MedChem Express) were set at 2.5  or 7.5 μmol·L^-1^. All 24/48-wells were photographed using a stereo microscope system (Leica EZ4 W).

### Bone resorption activity measurement

Bone slices were prepared from the cortical bones of bovine femurs pre-cleaned in 75% ethanol. A co-culture assay was initiated on these slices in 48-well plates. After 10 days, all cells were eliminated with ultrasound. The bone slices were mounted on carbon conductive tapes and photographed under a scanning electron microscope (FEI, Inspect F50) using 20-kV backscattered electrons.

### Western Blot analysis and co-immunoprecipitation assay

Total proteins were extracted from primary BMSC or OB cultures using a mammalian Protein extraction reagent (M-PER, Thermo Scientific), and the concentration was tested using a bicinchoninic kit (Beyotime Biotechnology). Electrophoresis was performed using 10% polyacrylamide gel and polyvinylidene fluoride (PVDF) membranes incubated overnight at 4 °C in 5% bovine serum albumin containing primary antibodies. The primary antibodies involved were as follows: anti-RUNX2 (Abcam, ab23981), anti-OSX (Abcam, ab22552), anti-NUMB (Abcam, ab14140), anti-HES1 (Abcam, ab108937), anti-PTEN (S380, Abcam, ab76431), anti-AKT (S473, Abcam, ab81283), anti-mTOR (Abcam, ab32028), anti-mTOR (S2448, Abcam, ab109268), anti-el4EBP41 (Abcam, ab32024), anti-el4EBP41 (T37, Abcam, ab75767), anti-GLI2 (Abcam, ab167389), anti–cAMP response element-binding (CREB; Abcam, ab32515), anti-pCREB (Abcam, ab32096), anti-AKT (Cell Signalling Technology, #4691), anti-AKT (T308, Cell Signalling Technology, #13038), anti-PTEN (Cell Signalling Technology, #9188), anti-NICD (Cell Signalling Technology, #4147), anti-S6 (Cell Signalling Technology, #2217), anti-S6 (S240/244, Cell Signalling Technology, #5364), anti-cleaved caspase-3 (Cell Signalling Technology, #9664), anti-GLI1, and anti-GLI3 (Santa Cruz Biotechnology, sc-20688). The secondary anti-rabbit/goat antibodies were purchased from Abcam. The protein content was normalized using mouse anti–glyceraldehyde 3-phosphate dehydrogenase (Abcam, ab8245), anti-alpha-tubulin (Abcam, ab52866), or anti-lamine B (Abcam, ab133741).

For the co-immunoprecipitation assay, primary osteoblasts were isolated from the calvaria of two-month-old C57B6J mice and cultured using a complete medium in 10-cm plates. The cells were harvested with 90% confluence, washed with cold PBS, and lysed using a cell lysis buffer for Western blotting and IP (Beyotime Biotechnology). Nonspecific binding proteins were eliminated by adding normal rabbit immunoglobulin G and protein A + G agarose. Target proteins were immunoprecipitated overnight at 4 °C using anti-NUMB (Abcam, ab14140) and anti-PTEN (Cell Signalling Technology, #9188). The samples were washed five times and analysed using Western blotting and anti-PTEN (Abcam, ab201856), anti-NUMB (Abcam, ab4147), and anti–neural precursor cell–expressed developmentally downregulated protein 4 (NEDD4; Abcam, ab188867) primary antibodies.

### Ubiquitination assay

The MC3T3-E1 cell line was purchased from the American Type Culture Collection. Plasmids encoding GFP-NUMB, GFP-m-Ub, FLAG-PTEN, FLAG-NEDD4-1, and related control plasmids were purchased from OriGene. DH5 competent cells (Tiangen), and TIANpure plasmid kit (Tiangen) were used to finish the transformation and purification of these plasmids. NEDD4-1 siRNA (OriGene) was used for knocking down the expression of *Nedd4-1*, and MG132 (MedChem Express) was used for inhibiting the proteasomes. Transfection was performed using Lipofectamine 3000 when MC3T3-E1 cells reached 80%–90% confluence. PTEN was immunoprecipitated using anti-FLAG (OriGene) or an anti-PTEN antibody (Cell Signalling Technology, #9188) 4 days after transfection. Then, polyacrylamide gel electrophoresis was used to separate the samples and an anti-ubiquitin (Abcam) antibody was used to detect ubiquitin.

### Enzyme-linked immunosorbent assay

Serum was collected from nine-week-old WT and DKO mice and stored at −80 °C. Enzyme-linked immunosorbent assay (ELISA) kits for acid phosphatase 5, tartrate-resistant acid phosphatase 5 (ACP5), osteocalcin (OCN), RANKL, and OPG were purchased from the Cloud Clone Corp. The results were read using a plate reader. Data were statistically analysed.

### Statistical analysis

The data were expressed as the mean ± standard deviation. Statistical significance was calculated using the Student paired *t* test. A *P* value <0.05 was considered statistically significant. The charts were prepared using GraphPad 5.0.

## Electronic supplementary material

Figure S1

Figure S2

Figure S3

Figure S4

Figure S5

Figure S6

Figure S7

Figure S8

Supplementary Materials
