# Supplementary figures and images for: NUMB maintains bone mass by promoting degradation of PTEN and GLI1 via ubiquitination in osteoblasts
Source: Bone Res. 2018 Nov 10;6:32. doi: 10.1038/s41413-018-0030-y (PMC6226489; doi:10.1038/s41413-018-0030-y)

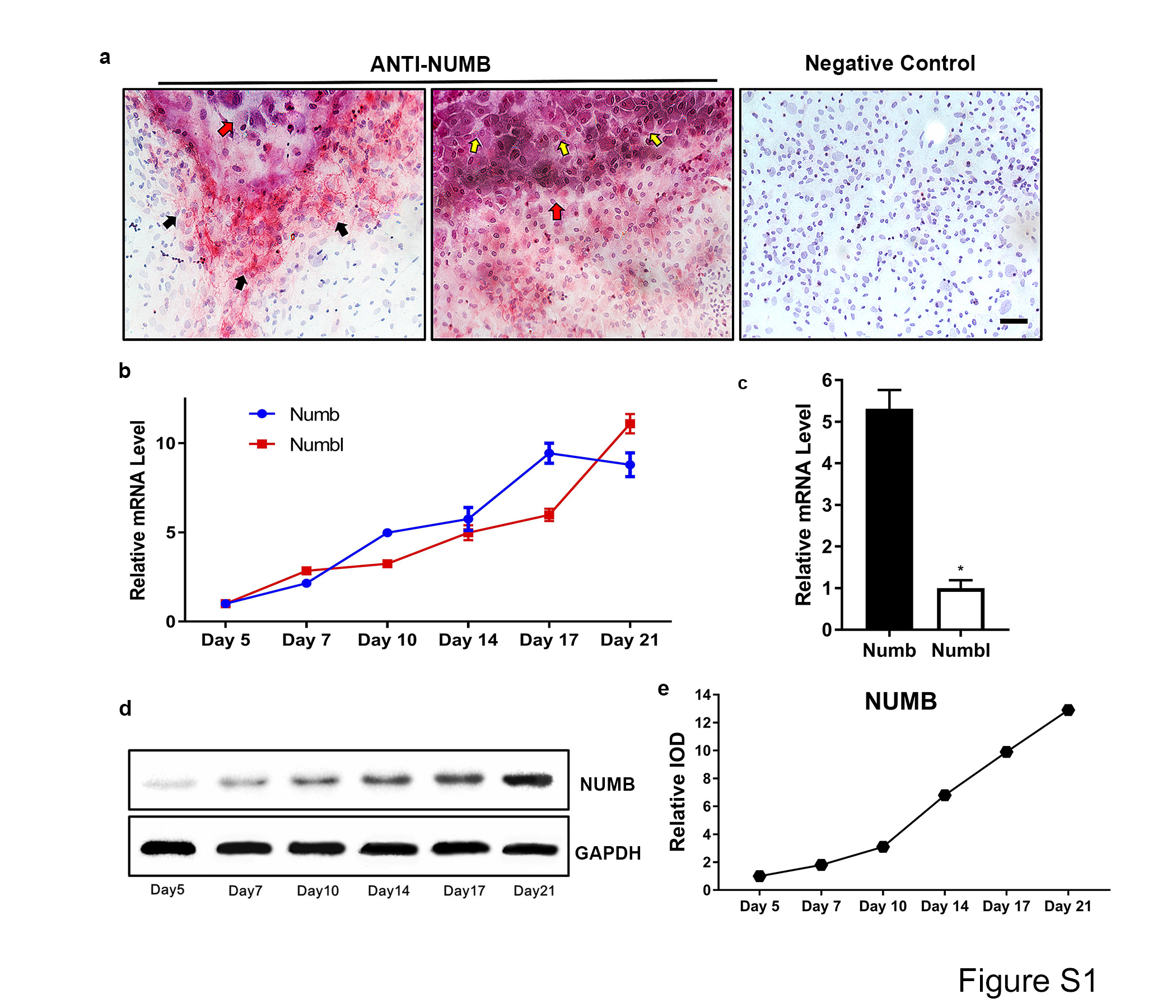

Supplement: Supplementary file 1 — Figure S1 [file 41413_2018_30_MOESM1_ESM.jpg]

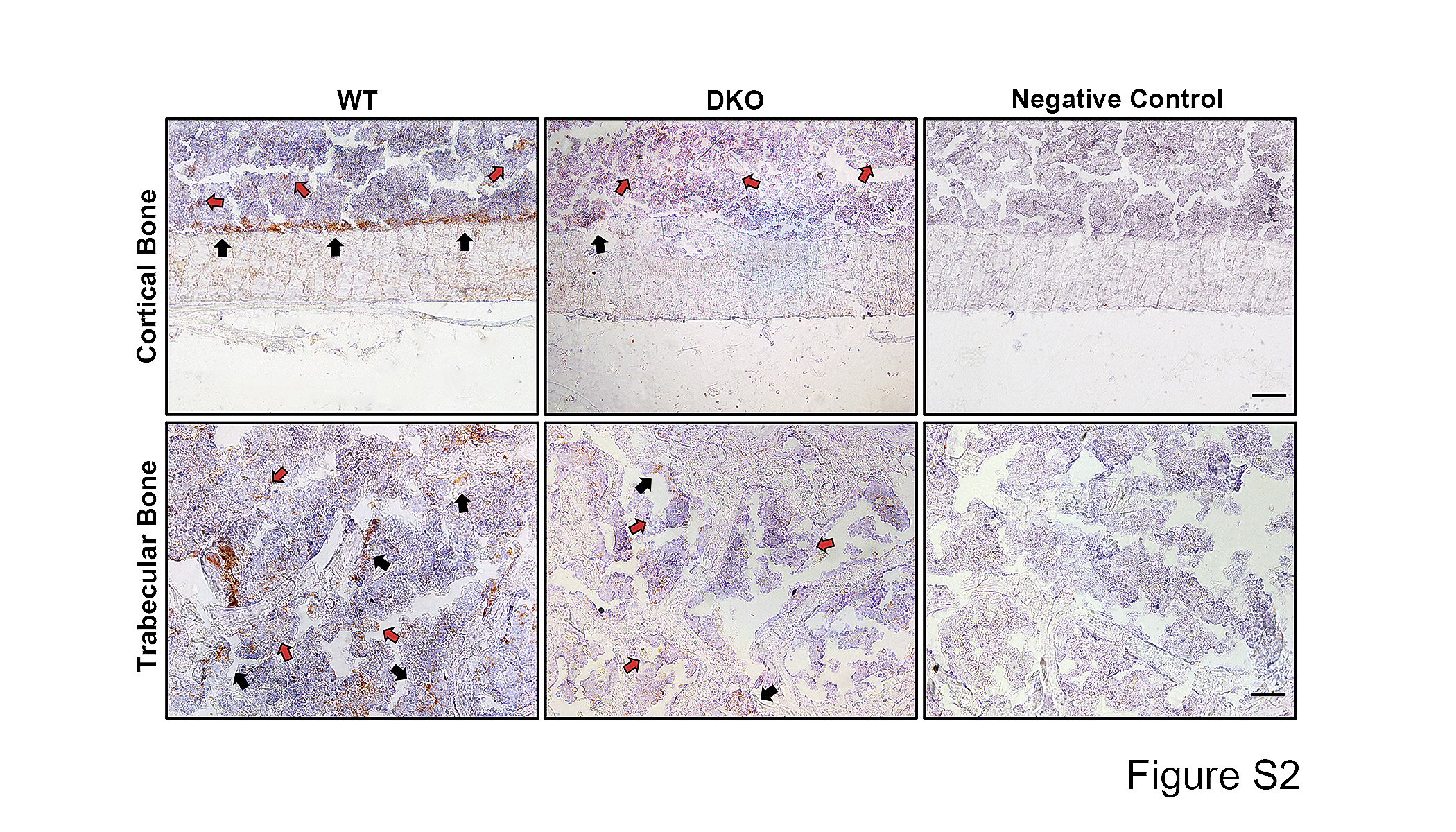

Supplement: Supplementary file 2 — Figure S2 [file 41413_2018_30_MOESM2_ESM.jpg]

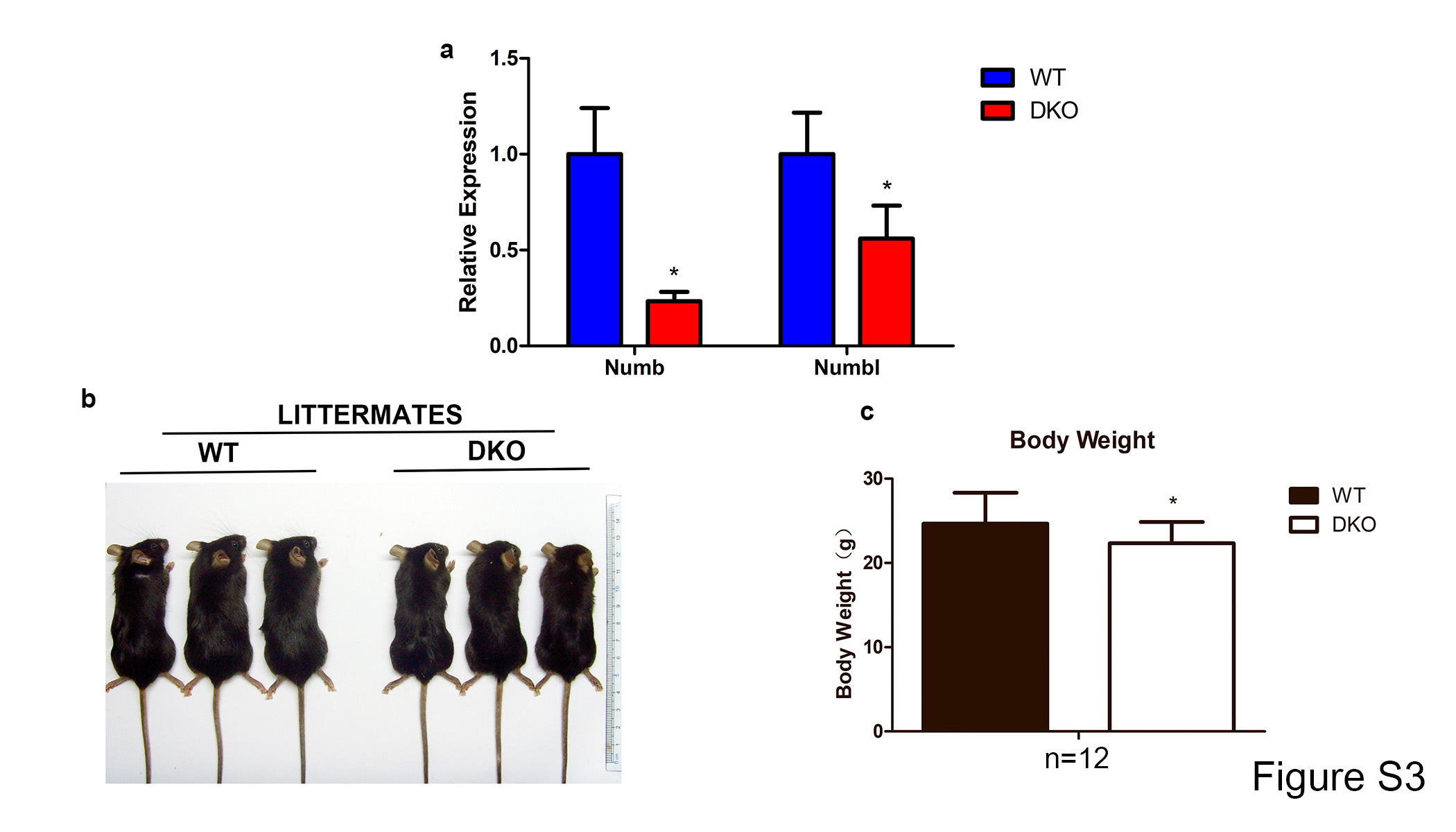

Supplement: Supplementary file 3 — Figure S3 [file 41413_2018_30_MOESM3_ESM.jpg]

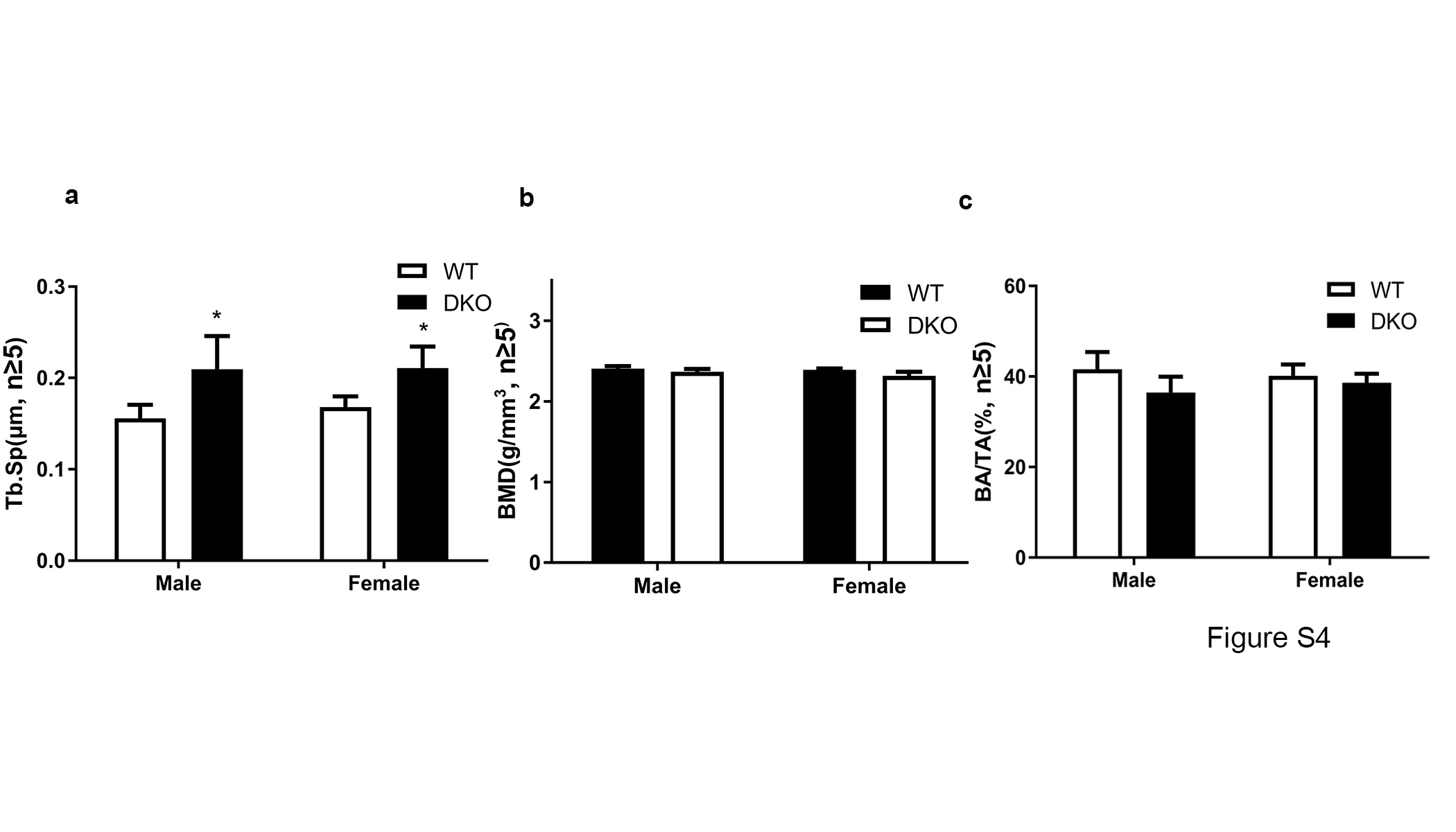

Supplement: Supplementary file 4 — Figure S4 [file 41413_2018_30_MOESM4_ESM.jpg]

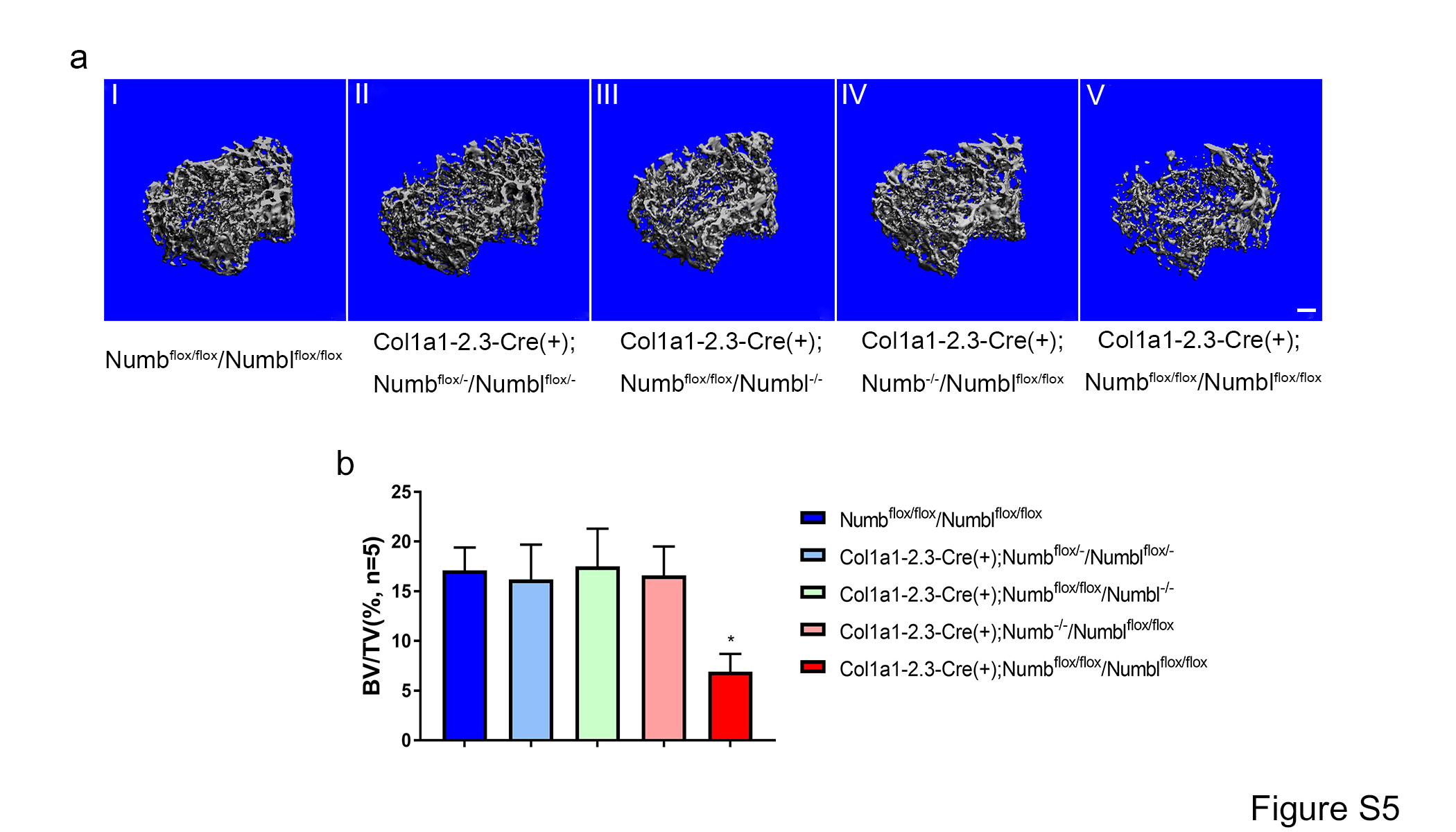

Supplement: Supplementary file 5 — Figure S5 [file 41413_2018_30_MOESM5_ESM.jpg]

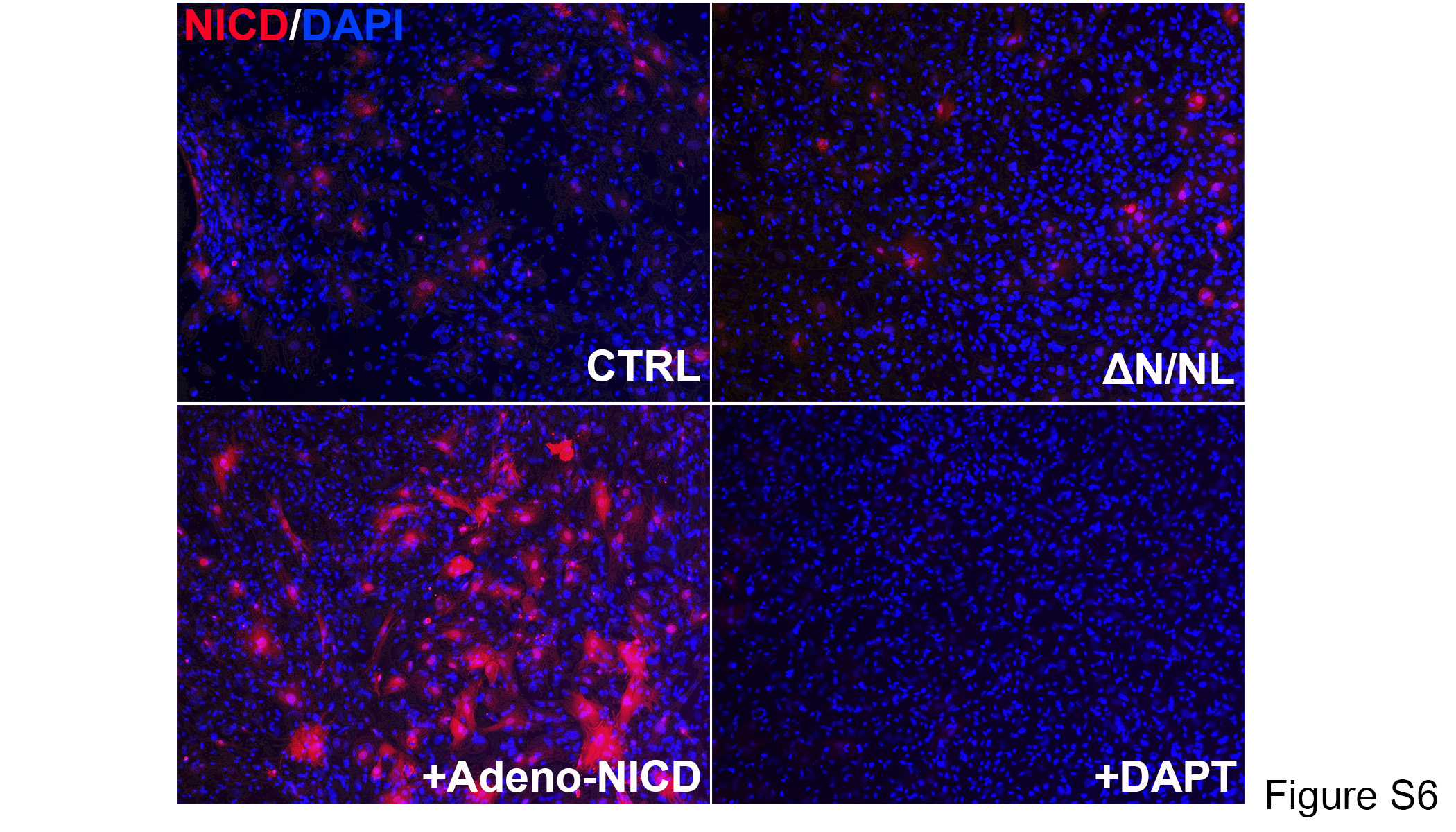

Supplement: Supplementary file 6 — Figure S6 [file 41413_2018_30_MOESM6_ESM.jpg]

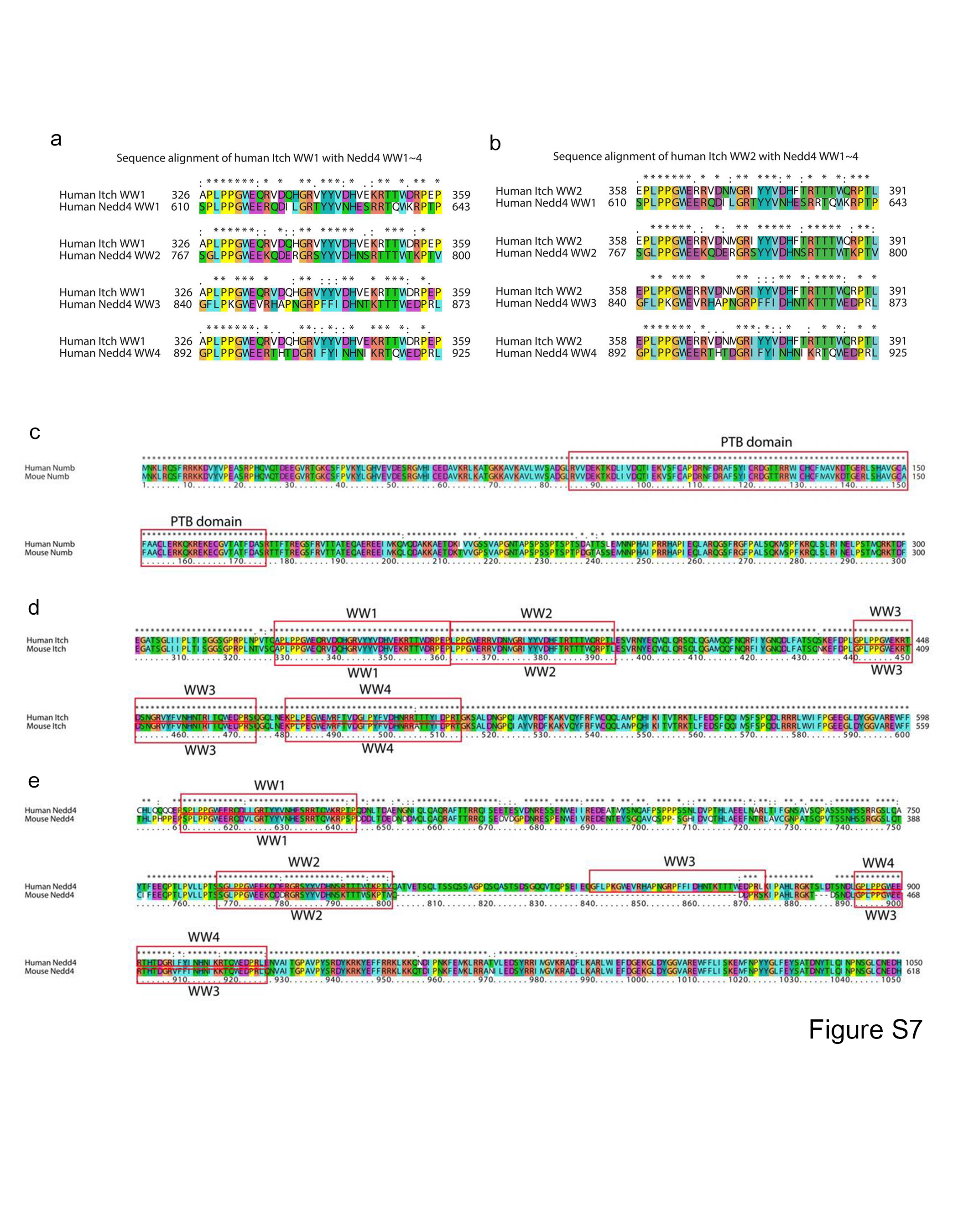

Supplement: Supplementary file 7 — Figure S7 [file 41413_2018_30_MOESM7_ESM.jpg]

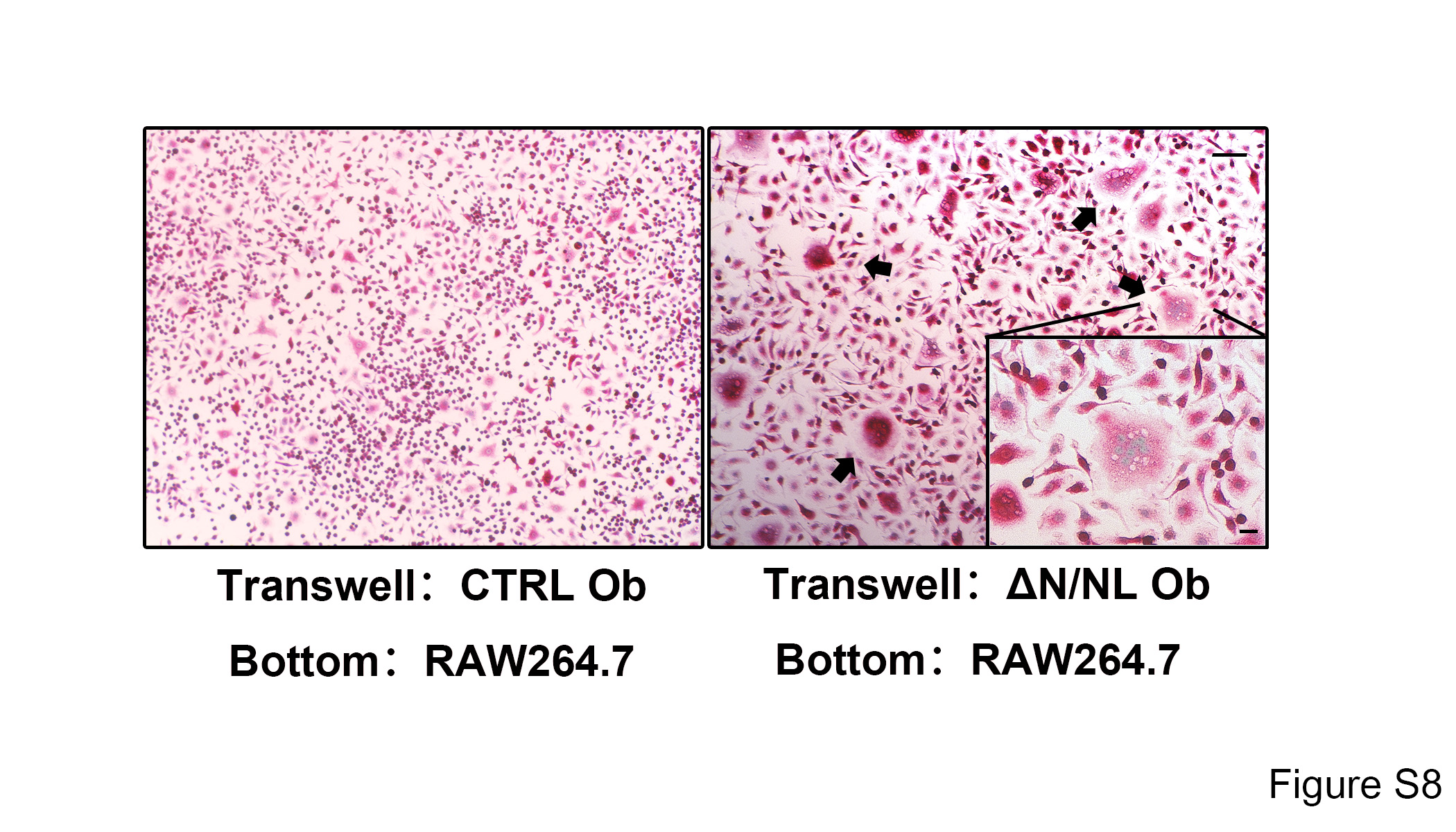

Supplement: Supplementary file 8 — Figure S8 [file 41413_2018_30_MOESM8_ESM.jpg]
