# Supplementary material for: NUMB maintains bone mass by promoting degradation of PTEN and GLI1 via ubiquitination in osteoblasts
Source: Bone Res. 2018 Nov 10;6:32. doi: 10.1038/s41413-018-0030-y (PMC6226489; doi:10.1038/s41413-018-0030-y)
Supplement: Supplementary file 9 — Supplementary Materials [file 41413_2018_30_MOESM9_ESM.docx]

**Supplemental materials**

**Figure S1. NUMB was highly expressed in osteoblastic lineage cells. BMSCs were isolated from femurs and stimulated to osteogenesis on day 7.** (a) On day 15, NUMB was immunostained with anti-NUMB (red), and the mineralized node (purple) was marked by haematoxylin. Osteoblast-like cells (black arrow), mineralizing node (red arrow), osteocyte-like cells (yellow arrow). Scale bar 50 μm. (b) RNA from BMSC cultures was extracted at six time-points (day 5, day 7, day 10, day14, day 17, and day 21). qRT-PCR was used to check both *Numb* and *Numbl* expression along with osteoblastic differentiation (c) and to check the relative expression between *Numb* and *Numbl* expression on day 21 (d). qRT-PCRs were normalized to β-actin. (e) Total protein was extracted on day 5, day 7, day 10, day 14, day 17, and day 21. Western blots were used to check NUMB protein expression. GAPDH was used for normalization and relative quantification (f). Data represent the mean ± SEM, *, P < 0.05.

**Figure S2. NUMB was located on the bone surface and 2.3-Cre effectively eliminated NUMB expression.** Immunohistochemistry was performed on frozen sections of 9-week-old WT and DKO femurs using anti-NUMB. The red arrow indicates a positive signal (brown) on the bone surface; the black arrow refers to a positive signal in the bone marrow. Scale bar, 100 μm.

**Figure S3. Gross phenotype of 9-week-old WT and DKO mice.** (a) Gene knockout of *Numb* and *Numbl* by 2.3-Cre is efficient. RNA was isolated from the femurs of 9-week-old WT and DKO mice and subjected to qRT-PCR analysis. Data were normalized to β-actin and represent the mean ± SEM, *, P < 0.05. (b) The gross appearance of DKO mice at 9 weeks. (c) A slight but significant decline in body weight was found in DKO mice (n=12). Data represent the mean ± SEM, *, P < 0.05.

**Figure S4. Supplement data on μCT analysis of DKO mice.** (a) Trabecular separation (Tb.Sp). (b) Bone mineral density (BMD). (c) Cortical bone fraction (BA/TA). Data shown represent the mean ± SEM, *, P < 0.05.

**Figure S5. μCT analysis of Numb/Numbl single knockout and heterozygous mice.** (a) Representative three-dimensional (3D) images of the trabecular bones in 9-week-old WT(Numb^flox/flox^/Numbl^flox/flox^, I), HET(Col1a1-2.3-Cre;Numb^flox/-^/Numbl^flox/-^, II), Numb KO(Col1a1-2.3-Cre;Numb^flox/flox^/Numbl^-/-^, III), Numbl KO(Col1a1-2.3-Cre;Numb^-/-^/Numbl^flox/flox^, IV) and DKO(Col1a1-2.3-Cre;Numb^flox/flox^/Numbl^flox/flox^, V) mice, scale bar, 200 μm. (b) Quantification of the bone volume fraction (BV/TV).

**Figure S6. Numb/Numbl deletion failed to change the cytosolic/nuclear NICD ratio.** Calvarial osteoblasts isolated from N/NL-floxed mice were transfected with adeno-GFP (CTRL) or adeno-Cre-GFP (ΔN/NL). Adeno-NICD-infected cells were used as positive controls, and DAPT chemically inhibits Notch signal levels as a negative control. NICD was marked red, and the nucleus was marked blue (DAPI). Scale bar, 100 μm.

**Figure S7 Sequence alignments** between the human Itch WW1 or WW2 domains (a) and the human Nedd4 WW1-4 domains (b). The sequence alignment of NUMB (a), ITCH (b) and NEDD4 (c) between human and mouse.“*”, highly conserved; “:”, moderately conserved, “.”, slightly conserved amino acid. Sequence similarity (SS) is the quotient between the number of conserved and total amino acids [(N_*_+ N_:_ + N_._)/ N_total_]. Sequence identity (SI) is the quotient between the number of highly conserved and total amino acids (N_*_/N_total_).

**Figure S8. ΔN/NL osteoblasts stimulate more multi-nuclei cells.** TRAP-stained RAW264.7 cells cultured on the lower transwell in 24-well plates on day 10. The upper transwell was cultured with ΔN/NL or CTRL osteoblasts. Black arrow: multi-nuclear cells. Scale bar, 100 μm. The small window shows several nuclei that (stained in blue). Scale bar, 20 μm.
